# Tumor-selective replication herpes simplex virus-based technology significantly improves clinical detection and prognostication of viable circulating tumor cells

**DOI:** 10.18632/oncotarget.9465

**Published:** 2016-05-18

**Authors:** Wen Zhang, Li Bao, Shaoxing Yang, Zhaoyang Qian, Mei Dong, Liyuan Yin, Qian Zhao, Keli Ge, Zhenling Deng, Jing Zhang, Fei Qi, Zhongxue An, Yuan Yu, Qingbo Wang, Renhua Wu, Fan Fan, Lianfeng Zhang, Xiping Chen, Yingjian Na, Lin Feng, Lingling liu, Yujie Zhu, Tiancheng Qin, Shuren Zhang, Youhui Zhang, Xiuqing Zhang, Jian Wang, Xin Yi, Liqun Zou, Hong-Wu Xin, Henrik J. Ditzel, Hongjun Gao, Kaitai Zhang, Binlei Liu, Shujun Cheng

**Affiliations:** ^1^ Department of Immunology, Cancer Hospital, Chinese Academy of Medical Sciences and Peking Union Medical College, Beijing 100021, China; ^2^ State Key Laboratory of Molecular Oncology, Cancer Hospital, Chinese Academy of Medical Sciences and Peking Union Medical College, Beijing 100021, China; ^3^ BGI-Shenzhen, Shenzhen 518083, China; ^4^ Department of Medical Oncology, Cancer Hospital, Chinese Academy of Medical Sciences and Peking Union Medical College, Beijing 100021, China; ^5^ Department of Medical Oncology, Cancer Center, West China Hospital of Sichuan University, Chengdu 610041, China; ^6^ Department of Pulmonary Oncology, Affiliated Hospital, Academy of Military Medical Science, Beijing 100071, China; ^7^ Department of Pathology, Cancer Hospital, Chinese Academy of Medical Sciences and Peking Union Medical College, Beijing 100021, China; ^8^ Department of Respiratory Diseases, Chinese PLA General Hospital, Beijing 100853, China; ^9^ Molecular Disease Biology Section, Department of Veterinary Disease Biology, Faculty of Health and Medical Sciences, University of Copenhagen, Danish Cancer Society, DK-2100 Copenhagen, Denmark; ^10^ Department of Cancer and Inflammation Research, Institute of Molecular Medicine, University of Southern Denmark, DK-5000 Odense C, Denmark; ^11^ The Guangdong Enterprise Key Laboratory of Human Disease Genomics, BGI-Shenzhen, Shenzhen 518083, China; ^12^ Shenzhen Key Laboratory of Transomics Biotechnologies, BGI-Shenzhen, 518083 Shenzhen, China; ^13^ James D. Watson Institute of Genome Science, 310008 Hangzhou, China; ^14^ Department of Oncology, Odense University Hospital, DK-5000 Odense C, Denmark; ^15^ Hubei Provincial Cooperative, Innovation Center of Industrial Fermentation, Hubei University of Technology, Wuhan 30068, China; ^16^ Institute of Laboratory Animal Science, Chinese Academy of Medical Sciences and Peking Union Medical College, Beijing 10021, China; ^17^ Institute of Environmental Health and Related Product Safety, China CDC, Beijing 10021, China; ^18^ Laborartory of Molecular Oncology, Key Laboratory of Carcinogenesis and Translational Research, Ministry of Education, Peking University Cancer Hospital and Institute, Beijing 100142, China; ^19^ Lung Cancer Center, Cancer Center, West China Hospital of Sichuan University, Chengdu 610041, China

**Keywords:** viable circulating tumor cells, epithelial-marker-independent, telomerase-specific HSV, clinical application

## Abstract

Detection of circulating tumor cells remains a significant challenge due to their vast physical and biological heterogeneity. We developed a cell-surface-marker-independent technology based on telomerase-specific, replication-selective oncolytic herpes-simplex-virus-1 that targets telomerase-reverse-transcriptase-positive cancer cells and expresses green-fluorescent-protein that identifies viable CTCs from a broad spectrum of malignancies. Our method recovered 75.5–87.2% of tumor cells spiked into healthy donor blood, as validated by different methods, including single cell sequencing. CTCs were detected in 59–100% of 326 blood samples from patients with 6 different solid organ carcinomas and lymphomas. Significantly, CTC-positive rates increased remarkably with tumor progression from N0M0, N+M0 to M1 in each of 5 tested cancers (lung, colon, liver, gastric and pancreatic cancer, and glioma). Among 21 non-small cell lung cancer cases in which CTC values were consecutively monitored, 81% showed treatment-related decreases, which was also found after treatments in the other solid tumors. Moreover, monitoring CTC values provided an efficient treatment response indicator in hematological malignancies. Compared to CellSearch, our method detected significantly higher positive rates in 40 NSCLC in all stages, including N0M0, N+M0 and M1, and was less affected by chemotherapy. This simple, robust and clinically-applicable technology detects viable CTCs from solid and hematopoietic malignancies in early to late stages, and significantly improves clinical detection and treatment prognostication.

## INTRODUCTION

Circulating tumor cells (CTCs) shed from the primary tumor site enter the circulation and may spread to other locations and initiate regional nodal or distant metastases [1, 2, 3, 4]. CTCs comprise a low percentage of circulating cells commonly present in cancer patients. With numbers as low as one CTC per 10^6^−10^7^ peripheral blood leukocytes, enrichment, investigation, and quantification of CTCs has proven extremely difficult [5], but the effort has been validated by current studies showing that the presence of CTCs in peripheral blood is a valuable prognostic marker in cancer patients [6, 7, 8, 9, 10, 11, 12], and an useful predictor of progression-free and overall survival. Results from clinical studies have shown breast, prostate and colorectal cancer patients with fewer CTCs in their peripheral blood survived longer than those with more CTCs [7, 8, 13]. Characterizing CTCs during drug treatment could also predict patient responses to specific anticancer drugs [14, 15, 16, 17], and some studies suggest that further investigation of CTCs could provide a clinically-relevant strategy to accurately determine cancer stage and/or treatment efficacy [18, 19]. There are currently a variety of methods to detect and quantify CTCs in peripheral blood [20, 21, 22], the majority of which are designed to identify the size of CTCs, antibodies against cell-surface molecules, or biological characteristics of cancer cells. Although these methods show some promise in detecting CTCs, they have limitations or are impractical for clinical use.

The most common current CTC identification techniques rely on antibodies against cell-surface molecules [20] using various antigens, such as EpCAM and cytokeratins (CKs), as markers for CTC detection or isolation. However, many studies have clearly shown that cell surface markers are different in different tumor cells [23, 24, 25], resulting in detection of only a subset of CTCs and subsequent underestimation of CTC numbers due to cell surface marker-negative CTC subsets [6, 26]. In addition, cell-surface marker-dependent approaches do not distinguish between viable or dead cells [27, 28]. Clearly, more sensitive, specific and reliable methods are needed for clinically meaningful application.

Measurement of CTC number during drug treatment could also predict the response to specific anticancer drugs [14, 15, 16, 17], and some studies suggest that CTC counts could be a clinically-relevant strategy to accurately determine cancer stage and/or treatment efficacy [18, 19]. Development of such methods will require further investigation into the source, biology and ultimate function of CTCs since their prognostic value has been verified only in a limited number of cancer types, e.g. breast, prostate and colorectal cancers.

An adenovirus system (OBP-401) for CTC detection based on biological characteristics of cancer cells has been reported [29], but while this system identified and enumerated viable CTCs, it had significant detection limitations. This system used a size threshold to define GFP-positive CTCs, but because tumor cells are heterogeneous in size [20, 30], OPB-401 may detect only a subset of larger CTCs. Moreover, OBP-401 failed to isolate CTCs by flow cytometry for subsequent single CTC analysis related to biologically-relevant mutations, new drug-resistance genes, new tumor markers, etc. Furthermore, the Ad-system cannot identify CTCs in patients with hematological malignancies. Adusumilli et al. [31] also reported the use of tumor-selective HSV expressing GFP to detect cancer cells in body fluids.

We previously reported the construction of an hTERT promoter-regulated oncolytic Herpes Simplex virus-1 (oHSV1-hTERT-GFP) in which the key transcriptional regulatory protein of HSV-1 was controlled by the hTERT promoter core sequence [32]. Here, we show that oHSV1-hTERT-GFP was strictly replicated in malignant tumor cells with detectable telomerase activity *in vivo* and *in vitro*, and this restricted replication did not cause lysis of the tumor cells within 48 hours post virus transduction. We further established a novel and highly applicable oHSV1-hTERT-GFP-based method to selectively label rare human CTCs in peripheral blood using GFP expression, which can be monitored by flow cytometry or fluorescence microscopy. This method not only allows specific detection and enumeration of viable CTCs independent of epithelial marker status, but may also permit molecular and genetic profiling of CTCs.

## RESULTS

### Labeling human cancer cells with oHSV1-hTERT-GFP

**Figure 1:**
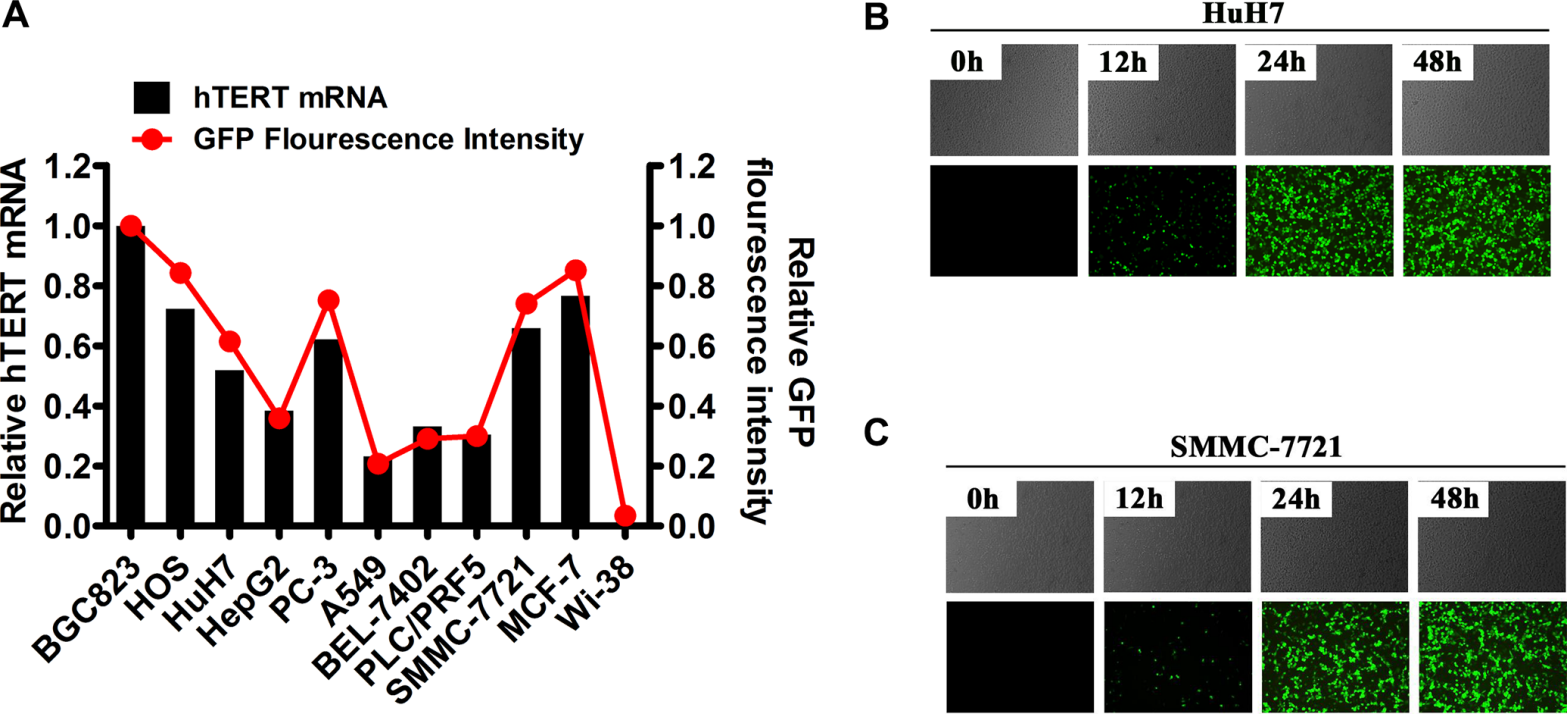
oHSV1-hTERTp-GFP selective replication in human cancer cells (**A**) The relative hTERT expression level and GFP fluorescence intensity in human tumor cell lines as determined by real-time RT-PCR analysis and flow cytometry, respectively. Relative hTERT mRNA expression ratios were normalized to the BGC823 cell line. Relative GFP fluorescence intensity was measured 48 hours after oHSV1-hTERTp-GFP transduction at an MOI of 0.1 and normalized to the BGC823 cell line. (**B**) Fluorescence micrographs showing GFP expression (bottom panels) of EpCAM+ HuH7 cells 48 hours after oHSV1-hTERTp-GFP transduction at an MOI of 1, and corresponding phase-contrast microscopy images of cell morphology (top panels). Original magnification, ×100. (**C**) Fluorescence micrographs showing GFP expression (bottom panels) of EpCAM-SMMC-7721 cells 48 hours after oHSV1-hTERTp-GFP transduction at an MOI of 1, and corresponding phase-contrast microscopy images of cell morphology (top panels). Original magnification, ×100.

To determine the correlation between hTERT and GFP expression in various human cancer cell lines following oHSV1-hTERT-GFP transfection, quantitative real-time polymerase chain reaction (qRT-PCR) for hTERT, and flow cytometry for GFP were performed in parallel. These data showed that the fluorescence intensity of GFP directly correlated with hTERT expressed in the various cancer cell lines (Figure 1A). GFP expression in EpCAM-positive or -negative cancer cell lines was evaluated, and both the EpCAM-positive human hepatocellular carcinoma cancer cell line, Huh7, and the EpCAM-negative hepatocellular carcinoma cancer cell line, SMMC-7721, exhibited GFP expression as early as 12 hours after oHSV1-hTERT-GFP transduction at Multiplicity of infection (MOI) = 1 (Figure 1B and 1C). Moreover, GFP fluorescence intensity increased over time, resulting in a strong GFP signal in nearly all cells 24 hours after transduction. In parallel experiments, oHSV1-hTERT-GFP also induced GFP expression in 7 other hTERT-positive cancer cell lines, including human gastric cancer cell BGC823, human lung cancer cell A549, human prostate cancer cell PC3, human osteosarcoma cell Hos, human glioma cell U251, human hepatocarcinoma cell BEL-7402, and HepG2, within 24 hours after transduction (Supplementary Figure 1).

### Elimination of oHSV1-hTERT-GFP-transduced white blood cells using fluorescent anti-CD45 antibody and FACS

**Figure 2:**
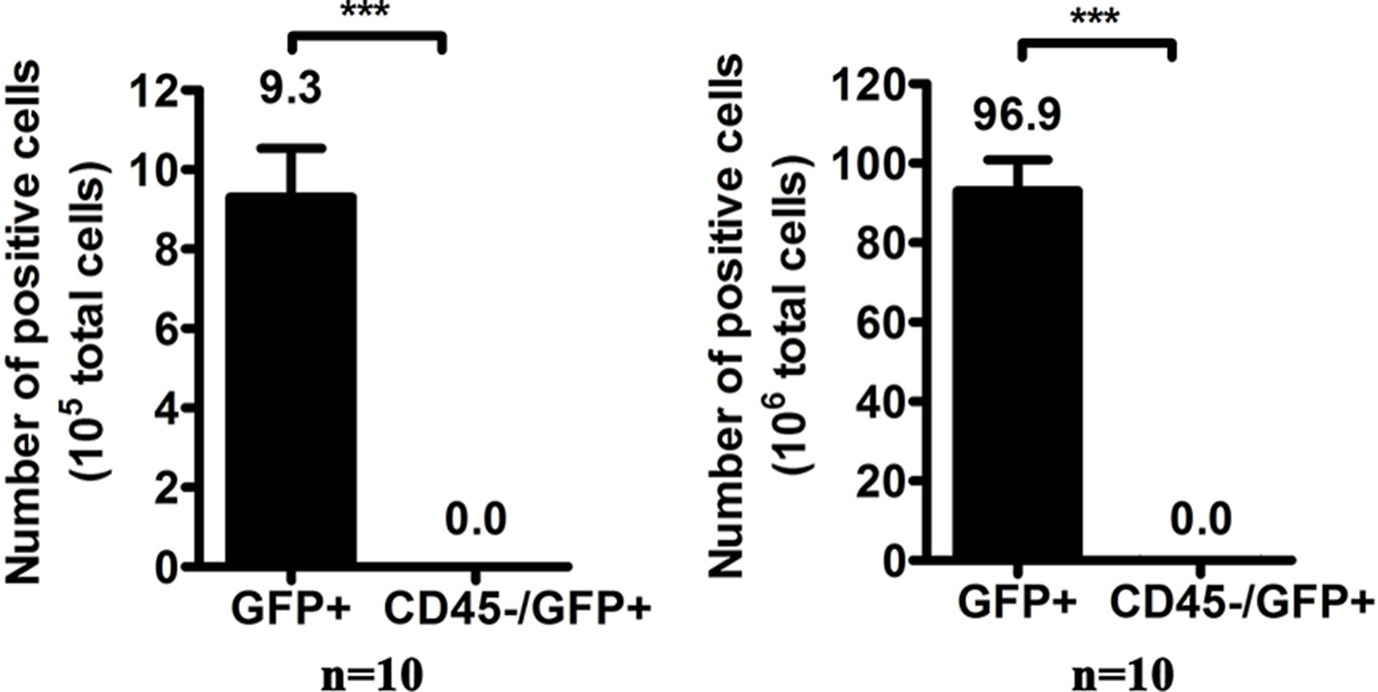
The number of GFP-positive cells per 10^6^ white blood cells after oHSV1-hTERT-GFP infection The columns marked GFP+ represent the total GFP+ cells investigated by our approach, and GFP+ cells include CD45+ and CD45−. Each value represents the mean ± SED of ten independent samples. ***p < 0.0001.

We noted in our preliminary investigation that a few white blood cells (WBCs) were transduced by oHSV1-hTERT-GFP, leading to the transient expression of GFP driven by the CMV promoter, which raised the concern that these GFP-positive (GFP+) WBCs might interfere with detection of tumor cells in the blood. Since all WBCs contain the CD45 cell surface marker, an anti-CD45 antibody was used in FACS analysis to remove all CD45-positive cells, including the virus-transduced GFP+ WBCs. Ten WBC samples from healthy donors were incubated with the virus at MOI = 1 for 24 hours followed by staining with a PE/Cy5-labeled CD45 antibody. FACS analysis revealed that the GFP+ cell numbers (including CD45+) were between 4–15 cells per 10^5^ WBCs and 47–133 cells per 10^6^ WBCs (*n* = 10), whereas the average CD45–/GFP+ cells were both 0.0 per 10^5^ WBCs and per 10^6^ WBCs (Figure 2). Therefore, the CD45–/GFP+ cells were used to represent CTCs throughout our investigation.

### Accuracy of the oHSV1-hTERT-GFP approach in various mimic CTC models

**Figure 3:**
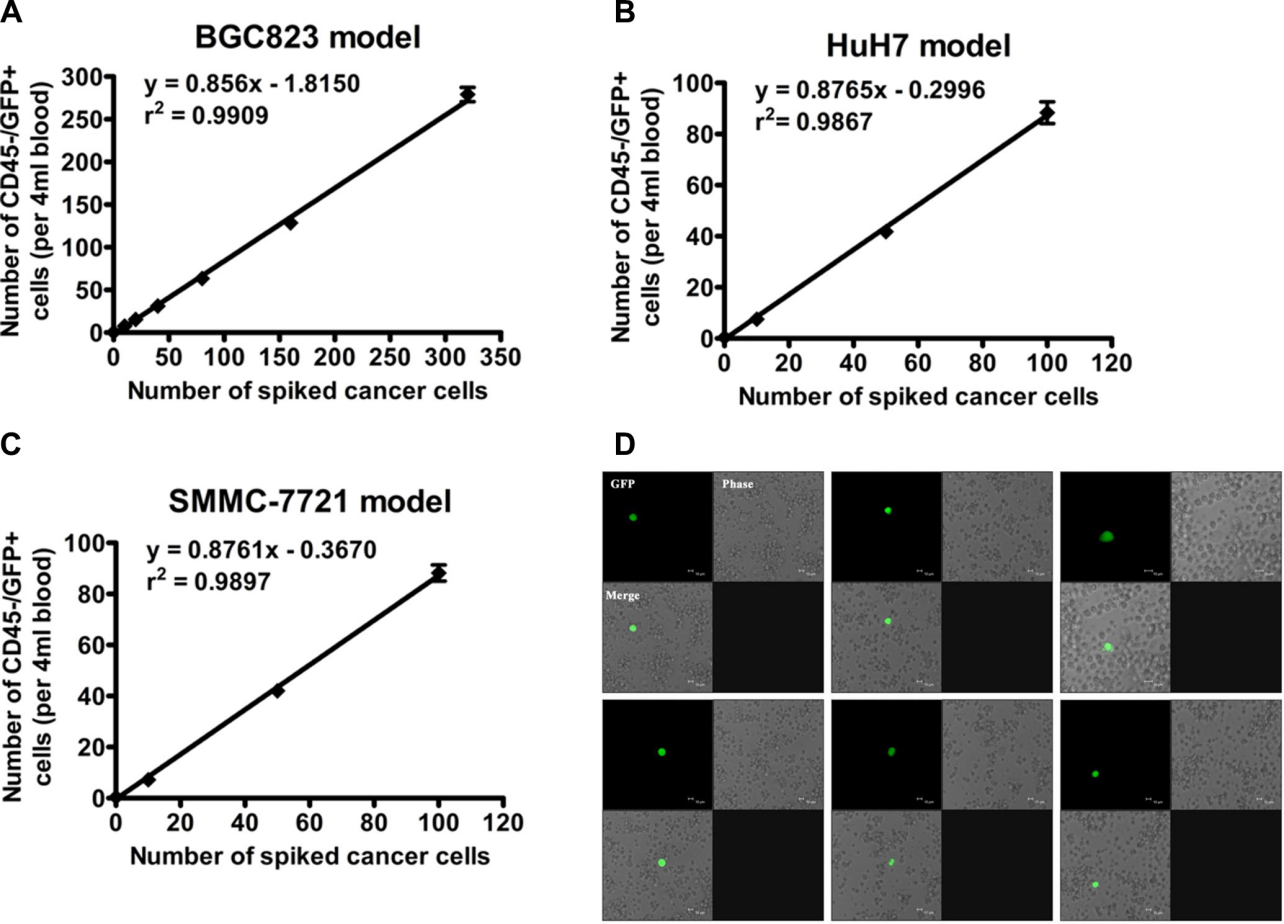
Accuracy of the oHSV1-hTERTp-GFP detection method Cancer cells (**A**) BGC823, (**B**) HuH7, and (**C**) SMMC-7721 were added to 4 ml of whole blood from healthy donors and identified by the oHSV1-hTERTp-GFP detection method. The number of cancer cells spiked into whole blood versus the number of GFP-expressing cells detected is plotted. The sample size of each point was listed in the Supplmentary Tables 1, 2, and 3. Each value represents the mean ± SD. (**D**) Typical mimic CTCs in the peripheral blood leukocytes of the blood sample spiked with 20 BGC823 cancer cells were treated with oHSV1-hTERTp-GFP. Typical spiked BGC823 cells were visualized using GFP expression by fluorescence microscope. Scale bar: 10 μm.

To test the efficacy and accuracy of the oHSV1-hTERT-GFP detection method, variable numbers of human cancer cell lines, including BGC823, Huh7 and SMMC-7721, were spiked into whole blood samples from healthy donors and analyzed using the oHSV1-hTERT-GFP replication methods. As shown in Figure 3A, the recovery rate of BGC823 cells was 75.5–87.2% over the frequency range of cancer cell numbers indicated (Supplementary Table 1). Regression analysis of the number of CD45−/GFP+ cells versus the number of spiked tumor cells showed a strong correlation (*r*^2^ = 0.9909). The HuH7 and SMMC-7721 cell tests also exhibited correlation efficiencies of *r*^2^ = 0.9876 and 0.9897, respectively (Figure 3B and 3C, Supplementary Tables 2 and 3). The GFP+ cells representing the spiked tumor cells were also visualized by fluorescence microscopy (Figure 3D), and the results suggest that the number of CD45–/GFP+ cells detected by our method was clinically relevant and reflected numbers of CTCs expected in peripheral blood.

### Validation of the origin of the CD45–/GFP+ cells by single-cell sequencing

**Figure 4:**
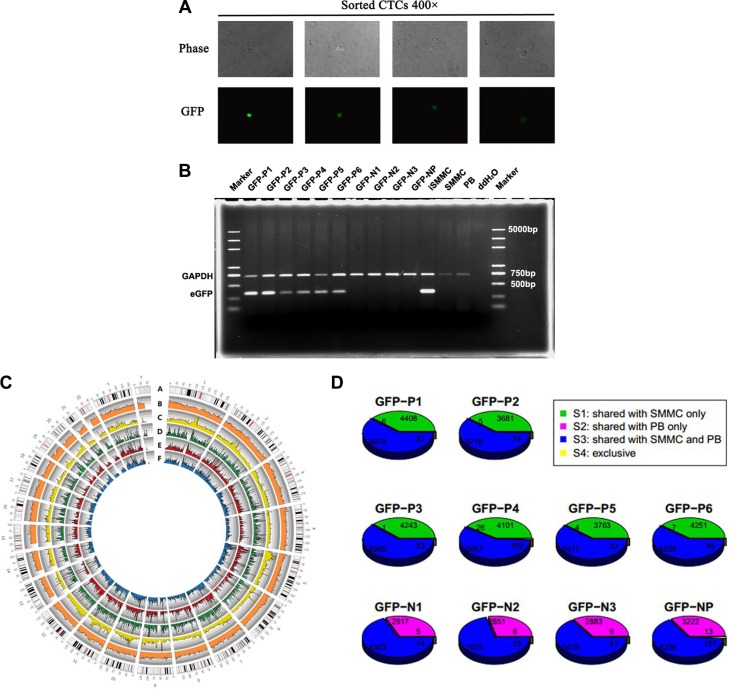
Validation by single-cell sequencing (**A**) Single CTCs sorted from peripheral blood samples from healthy donor spiked with 20 of SMMC-7721 cancer cells and observed by fluorescence microscopy. Each panel shows the fields from separate sorts. Original magnification, ×200. (**B**) The eGFP gene PCR products were analyzed by agarose gel electrophoresis. The order of samples: Marker, GFP-P1, GFP-P2, GFP-P3, GFP-P4, GFP-P5, GFP-P6, GFP-N1, GFP-N2, GFP-N3, GFP-NP, transduced SMMC-7721 cell line (iSMMC, positive control), non-transduced SMMC-7721 cell line (SMMC, negative control), peripheral blood cells of a healthy donor (PB, negative control), ddH_2_O, and Marker. Upper: GAPDH, lower: eGFP. (**C**) Circos map of the whole exomes of the unamplified bulk DNA, the pooled cells and 2 single SMMC-7721 cells: A. Karyotype of the human reference genome (Hg19); B. GC content distribution of the whole exome (the heights of the orange rectangles ranged from 0–100%, and the whole exome was divided based on 1 Mb bins of the reference genome); C. Whole-exome depth distribution of the unamplified bulk DNA of the SMMC-7721 cell line (the heights of the yellow rectangles ranged from 0–160X, the whole exome was divided based on 1 Mb bins of the reference genome); D. Whole-exome depth distribution of the pooled cells GFP-NP (the heights of the green rectangles ranged from 0–80X, and the whole exome was divided based on 1 Mb bins of the reference genome); E. Whole-exome depth distribution of cell GFP-P2 (the heights of the red rectangles ranged from 0–20X, and the whole exome was divided based on 1 Mb bins of the reference genome); and F. Whole-exome depth distribution of cell GFP-P3 (the heights of the blue rectangles ranged from 0–20X, and the whole exome was divided based on 1 Mb bins of the reference genome). (**D**) Comparison of single nucleotide polymorphisms (SNPs) of the amplified DNA products of single-cell samples with unamplified bulk DNA of the SMMC-7721 cell line and peripheral blood cells of a healthy donor: SNPs of each sample were separated into 4 subsets (S1: the green section, consistent with the SMMC-7721 cell line only, S2: the pink section, consistent with peripheral blood cells of the healthy donor only, S3: the blue section, consistent with both the SMMC-7721 cell line and peripheral blood cells, and S4: the yellow section, not consistent with either the SMMC-7721 cell line or peripheral blood cells). Numbers of SNPs belonged to the subsets of each sample were marked on the pie charts. The upper 6 single cells belonged to the GFP-positive population, whereas the lower 3 single cells and the cell pool belonged to the GFP-negative population.

To confirm that oHSV1-hTERT-GFP-labeled cells were cancer cells and not normal cells, SMMC-7721 cancer cells were spiked into a peripheral blood sample from a healthy donor, exposed to oHSV1-hTERT-GFP, and CD45–/GFP+ and CD45+/GFP– cells isolated (Figure 4A). Six single cells from the CD45–/GFP+ population, three single cells and one pooled cell from the CD45+/GFP– population, were whole-genome amplified and the product examined. The results showed that a single band was obtained only from PCR products containing sufficient amounts of oHSV1-hTERT-GFP DNA. Furthermore, Sanger sequencing showed that the PCR-positive products matched the DNA sequence of oHSV1-hTERT-GFP, which implies that all 6 CD45–/GFP+ single cells efficiently replicated oHSV1-hTERT-GFP, while none of the 3 CD45+/GFP– single cells or the CD45+/GFP– cell pool were capable of replicating the intracellular virus (Figure 4B).

Next, we sequenced the amplification products of the 9 single cells at a depth of ~5× as well as the unamplified bulk DNA of the SMMC-7721 cell line and the peripheral blood of the healthy donor at ~50× depth and pooled cells at ~20× depth, and evaluated the whole exome recovery and amplification uniformity of single-cell sequencing using a Circosmap [33]. The mean coverage rate (≥ 1×) of single-cell sequencing in the exome regions was 70.62% (range 52.50–87.03%) at a 6.6× mean sequencing depth (Supplementary Table 4). According to the sequence recovery and distribution uniformity of the whole exome, the single-cell sequencing data showed uniform whole-exome coverage, highly similar to that of the unamplified bulk (Figure 4C).

After single nucleotide polymorphisms (SNPs) calling, the SNPs of each sample were separated into 4 subsets (S1–4) that clearly distinguished the source of the single cells (Figure 4D). Single-nucleotide substitutions of the 6 GFP+ single cells were consistent with the whole exome data of the bulk DNA of the SMMC-7721 cell line, and at least 98.5%, of the SNPs were shared, whereas the SNPs of 3 GFP− single cells and the GFP− cell pool were consistent with those of peripheral blood cells of the healthy donor. These findings indicated that the 6 GFP+ single cells were derived from the SMMC-7721 cell line, and the 3 GFP-single cells and 1 GFP− pooled cells were derived from peripheral blood cells of the healthy donor.

Moreover, the SNPs of the S2 subset of the 6 GFP+ single cells probably belonged to the shared SNPs (S3 subset), but were not detected in the sequencing data of the bulk DNA of the SMMC-7721 cell line due to the different coverage of every SNP locus, as well as those of the S1 subset of the 3 GFP− single cells and the GFP-cell pool. The S4 subset may represent specific mutations of each sample or false-positive calling of SNPs due to errors in whole genome amplification.

### Identification of CTCs in patients with solid tumors and hematological malignancies

**Figure 5:**
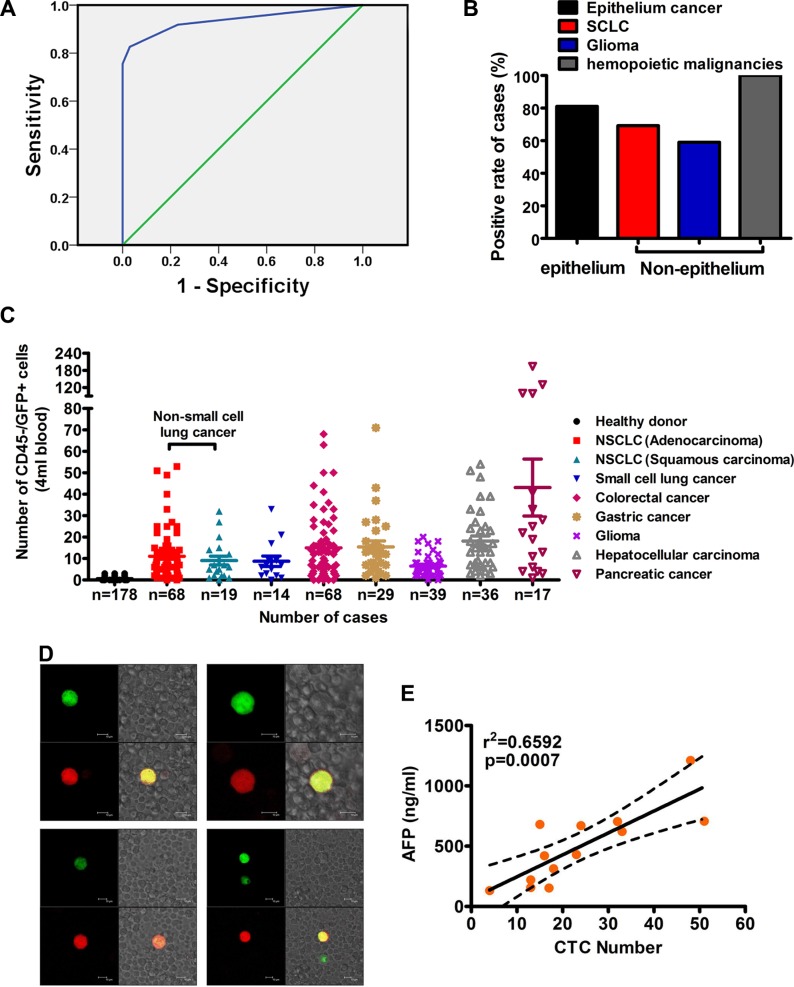
Identification of CTCs in patients with solid tumors (**A**) ROC analysis for threshold detection of CTCs. The threshold was determine by compared patients and controls by ROC analysis, identifying the threshold to 3 cells, and consequently measurements of > 3 CD45–/GFP+ cells was considered positive. A significant difference between patients and controls was observed (*p* < 0.001; area under the curve (AUC) = 0.937; 95% confidence interval = 0.914–0.960). (**B**) Positive rate for epithelial and non-epithelial cancer, including SCLC, glioma, and lymphoma. (**C**) Enumeration of CTCs in peripheral blood of solid tumor patients. CTC counts in 4-ml blood samples from 290 patients with solid cancers, 68 with NSCLC adenocarcinoma, 19 with NSCLC squamous carcinoma, 14 with small cell lung cancer, 68 with colorectal cancer, 29 with gastric cancer, 39 with glioma, 36 with hepatocellular carcinoma, and 17 with pancreatic cancer. Approximately 1 × 10^7^ cells were counted in 4 ml of blood from patients with solid tumors. (**D**) Typical CTCs in the peripheral blood leukocytes of the blood sample were visualized using GFP expression. The blood samples were incubated with oHSV1-hTERT-GFP, then stained with Alexa Fluor^®^ 647-labeled anti-CK18 antibody. Overlap of green (GFP) and red (CK18) fluorescence was displayed as yellow fluorescence. Approximately 80% of GFP+ cells were CK18+. Scale bar, 10 μm. (**E**) Correlation of CTCs and AFP in HCC patients (*n* = 13, the best-fit line and 95% confidence bands are indicated). Each dot represents an individual blood sample; 95% confidence interval, 9.500 to 26.84.

To determine whether CTCs from patients with solid tumors could be detected by oHSV1-hTERT-GFP, we analyzed 290 whole blood samples of patients with various cancer types. The positive threshold of > 3 CTCs per 4 ml blood was calculated based on ROC analysis (Figure 5A) of the CTC data from 178 healthy donors and 290 solid tumor patients. CTCs from patients with both epithelial and non-epithelial solid tumors (Figure 5B) were detected, including 52 of 68 non-small cell lung cancer (NSCLC) adenocarcinomas (NSCLC-AC) (76.5%), 16 of 19 NSCLC squamous cell carcinomas (NSCLC-SC) (84.2%), 9 of 14 small cell lung cancer (SCLC) (69.2%), 51 of 68 colorectal cancers (CC) (75%), 25 of 29 gastric carcinomas (GC) (86.2%), 23 of 39 gliomas (59%), 33 of 36 hepatocellular carcinomas (HCC) (91.6%), and 15 of 17 pancreatic cancers (PC)(88.2%) (Supplementary Tables 5–11). The average numbers of detected CD45−/GFP+ cells were 11.1, 9.1, 8.7, 15.0, 15.5, 6.5, 18.2, and 43.1 for NSCLC-AD, NSCLC-SC, SCLC, CC, GC, glioma, HCC, and PC patients, respectively (Figure 5C). The average numbers of CD45−/GFP+ cells were 0.63 for healthy donor, while average CTC number for cancer patients was 10 to 68-fold higher. Although CTCs are significantly elevated on average, several patients with different cancer types did not have detection of CTC (≤ 3 cells).

The identity of the CTCs from non-small cell lung cancer patients isolated using our approach was validated by staining for the epithelial marker CK18. Representative confocal microscopy images are shown in Figure 5D. The cells that were identified as CTCs by their GFP expression were also marked by the CK18 antibody. Since the number of CTCs in HCC has been correlated with levels of the alpha-fetoprotein (AFP) HCC marker [34] (Schulze, Gasch et al. 2013), we performed similar comparison assessments. Thirteen of 36 HCC patients expressed AFP above the cut-off > 100 ng/ml. All these patients were also CTC-positive by oHSV1-hTERT-GFP detection, and a positive correlation between CTC numbers and AFP levels was observed (*r*^2^ = 0.6592, *p* = 0.007) (Figure 5E). These findings suggested that the oHSV1-hTERT-GFP method can reliably detect CTCs in clinical practice. In agreement with other studies [35], CTCs were also detected in the peripheral blood of patients with primary gliomas (Figure 5B). In addition, abnormal cells that were most likely disseminated tumor cells (DTCs) were observed in cerebrospinal fluid (Supplementary Figure 2).

To determine whether this approach could also detect CTCs in patients with hematological malignancies, the number of GFP+ cells in blood samples of patients and healthy donors treated with oHSV1-hTERTp-GFP were analyzed. As shown in Supplementary Figure 3, the average GFP+ cells were 8.5 (4–15) per 10^5^ total cells in control group (*n* = 100). In contrast, the GFP+ cells increased to a high level ranging from 19 to 687 per 10^5^ total cells for Non-Hodgkin's lymphoma (*n* = 14), 46–2767 per 10^5^ total cells for natural killer/T cell Non-Hodgkin's lymphoma (NK/T) (*n* = 11), 121–807 per 10^5^ total cells for diffuse large B-cell lymphoma (*n* = 9), and 343–609 per 10^5^ total cells for Hodgkin's lymphoma (*n* = 2) (Supplementary Table 12).

### Positive CTC rates correlate well with cancer staging

**Figure 6:**
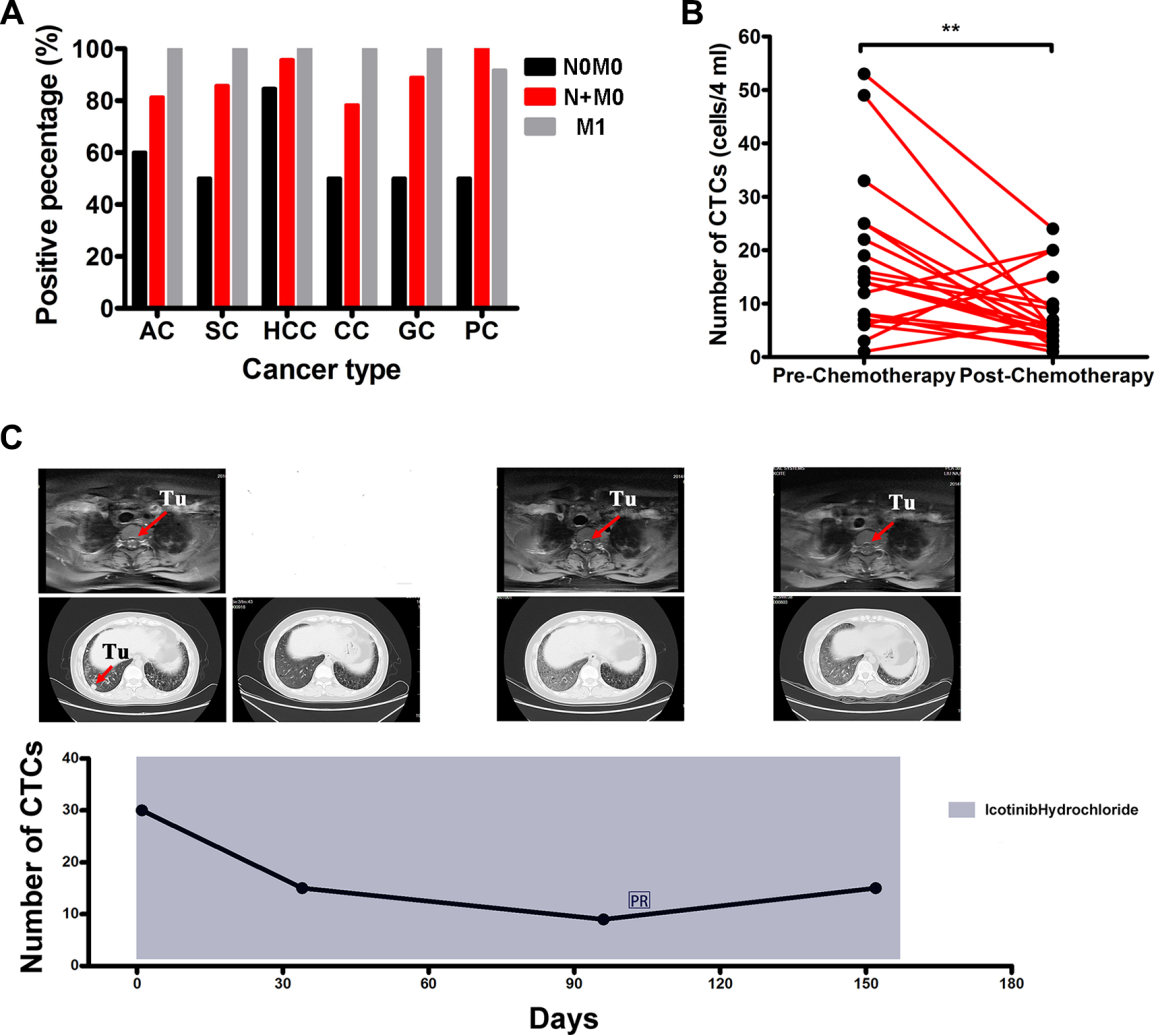

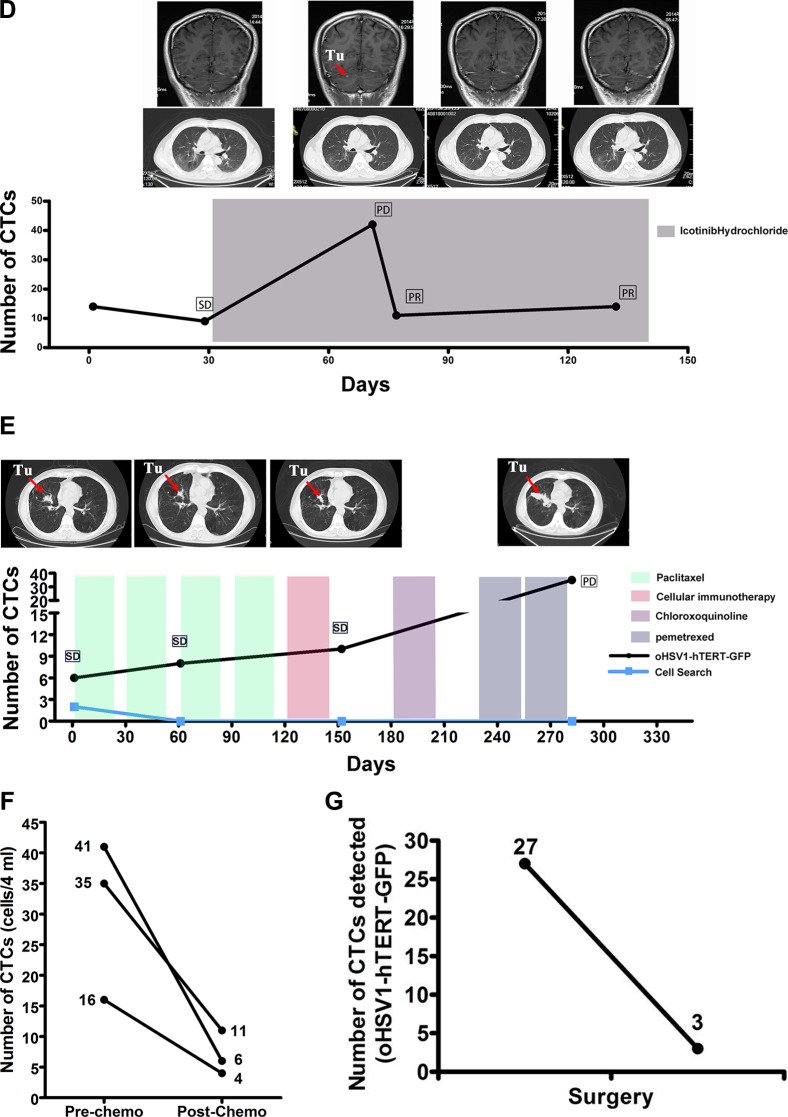
Correlations between CTC-positive rates and cancer stage or treatment response (**A**) Positive rates of CTC numbers for patients with cancer at different TNM stages detected by oHSV1-hTERT-GFP. AC: adenocarcinoma (NSCLC); SC: squamous carcinoma (NSCLC); HCC: hepatocellular carcinoma; CC: colorectal cancer; GC: gastric carcinoma; PC: pancreatic cancer. (**B**) Detection of patients with non-small cell lung cancer (adenocarcinoma) and their CTC numbers during or after chemotherapy. **p = 0.0056. (**C**) Changes in CTC numbers in response to treatment in a patient with stage III NSCLC using oHSV-hTERT-GFP. The numbers of CTCs were 30, 15, 9, and 15 at different time points, respectively. Upper: spine CT cross-sectional images, Middle: chest CT cross-sectional images, Tu: tumor. (**D**) CTC changes in response to treatment in a patient with stage III NSCLC using oHSV-hTERT-GFP. The numbers of CTCs were 9, 42, 11, and 14 at different time points, respectively. Upper: brain CT cross-sectional images, Middle: chest CT cross-sectional images, Tu: tumor. (**E**) CTC changes in response to treatment in a patient with stage III NSCLC using oHSV-hTERT-GFP or CellSearch. The numbers of CTCs were 6, 8, 10, and 36 at different time points for oHSV1-hTERT-GFP, respectively, and 2, 0, 0, and 0 at different time points for CellSearch, respectively. Upper: chest CT cross-sectional images, Tu: tumor. (**F**) Changes in CTC numbers after chemotherapy of three colorectal cancer cases. (**G**) Changes in CTC numbers after surgery of colorectal cancer cases.

To validate the sensitivity of the oHSV1-hTERT-GFP detection method, positive CTC rates from 186 blood samples from patients with cancers at different stages were analyzed. As shown in Figure 6A and Tables 1 and 2, the CTC positive rates of patients with regional lymph node metastasis as determined by pathology, but without distant metastasis (N+M0) were 81.3% for NSCLC-AD (37 of 48), 85.7% for NSCLC-SC (6 of 7), 95.7% of HCC (22 of 23), 77.4% for colon cancer (24 of 31), 88.9% for gastric cancer (24 of 27), and 100% for pancreatic cancer (11 of 11), respectively. For patients with metastases to distant organs, the positive rates were 100% for all but pancreatic carcinoma (91.7%). Interestingly, 50%−60% positive CTC rates were observed for patients with biopsy-verified invasive malignancy at site of origin, but without regional nodal or distant metastasis (N0M0), suggesting that some N0M0 patients might have nodal or distant micro-metastases that could not be detected by standard assessment. Similarly, the positive rate of CTCs in gliomas rose progressively with advancing stage of disease (Table 2). No statistical difference was observed between the percentages in stage M1 and N+M0 (Figure 6A).

**Table 1:** Detection of CTCs in lung cancer patients using oHSV1-hTERT-GFP

Type	TNM or stage	Number of patients with > 3 CTCs	Total number of patients	Sensitivity^*^
Adenocarcinoma	T1N0M0	3	4	0.75
	N0M0	12	20	0.6
	N + M0	37	48	0.813
	M1	15	15	1
Squamous cell carcinoma	N0 M0	2	4	0.5
	N + M0	6	7	0.857
	M1	5	5	1
Small cell lung cancer	LS	1	4	0.25
	ES	8	10	0.8

Note:

* Sensitivity = Number of patients with > 3 CTCs/Total number of patients. ES, Extensive stage; LS, Limited stage.

**Table 2:** Detection of CTCs in patients with various cancer types using oHSV1-hTERT-GFP

Type	TNM or Grade	Number of patients with > 3 CTCs	Total number of patients	Sensitivity^*^
Hepatocarcinoma	N0 M0	11	13	0.846
	N+ M0	22	23	0.957
	M1	12	12	1
Colon carcinoma	N0 M0	4	8	0.5
	N + M0	47	60	0.783
	M1	22	22	1
Gastric carcinoma	N0 M0	1	2	0.5
	N + M0	24	27	0.889
	M1	8	8	1
Pancreatic carcinoma	N0 M0	3	6	0.5
	N + M0	11	11	1
	M1	11	12	0.917
Glioma	II	11	23	0.478
	III	9	13	0.692
	IV	12	15	0.8

Note:

* Sensitivity = Number of patients with >3 CTCs / Total number of patients.

### Monitoring CTC counts following treatment of NSCLC patients and hematological malignancies

To evaluate the possibility of monitoring treatment responses in patients with solid organ malignancies using our approach, we further assessed CTC dynamics in NSCLC patients (*n* = 21) undergoing chemotherapy. As shown in Figure 6B, among 21 NSCLC cases in which CTC values were consecutively monitored, 81.0% (17 cases) showed decreased CTCs numbers in those with favorable treatment responses (Supplementary Table 13). A significant reduction in CTC pre- and post-treatment was observed (*p* = 0.0056). Three representative NSCLC cases with reduced CTC numbers in response to treatment are shown in Figure 6C, 6D and 6E. The changes in CTCs numbers correlated well with clinical diagnosis and imaging. In 4 image traceable colon cancer patients, three being treated with chemotherapy and one undergoing surgery, CTC numbers decreased 4 weeks after treatments (Figure 6F and 6G).

In addition, CTC numbers in 6 follow-up lymphoma patients after two cycles of chemotherapy all showed decreased average CTC counts, and 4 of which were significant. Moreover, the reduction in CTCs correlated well with the clinical evaluation of apparently curative effect (Supplementary Figure 4). The results from a representative patient (case 6) with non-Hodgkin's lymphoma are shown in Supplementary Figure 4. The patient recrudesced 90 days after chemotherapy, and the average number of CTCs per 10^5^ rose from 350 to 1,354. These results suggest that the number of CTCs could be useful for monitoring clinical treatment response and disease recurrence in patients with solid organ epithelial cancers or hematological malignancies.

### Comparison of CTC detection using oHSV1-hTERT-GFP and CellSearch

**Figure 7:**
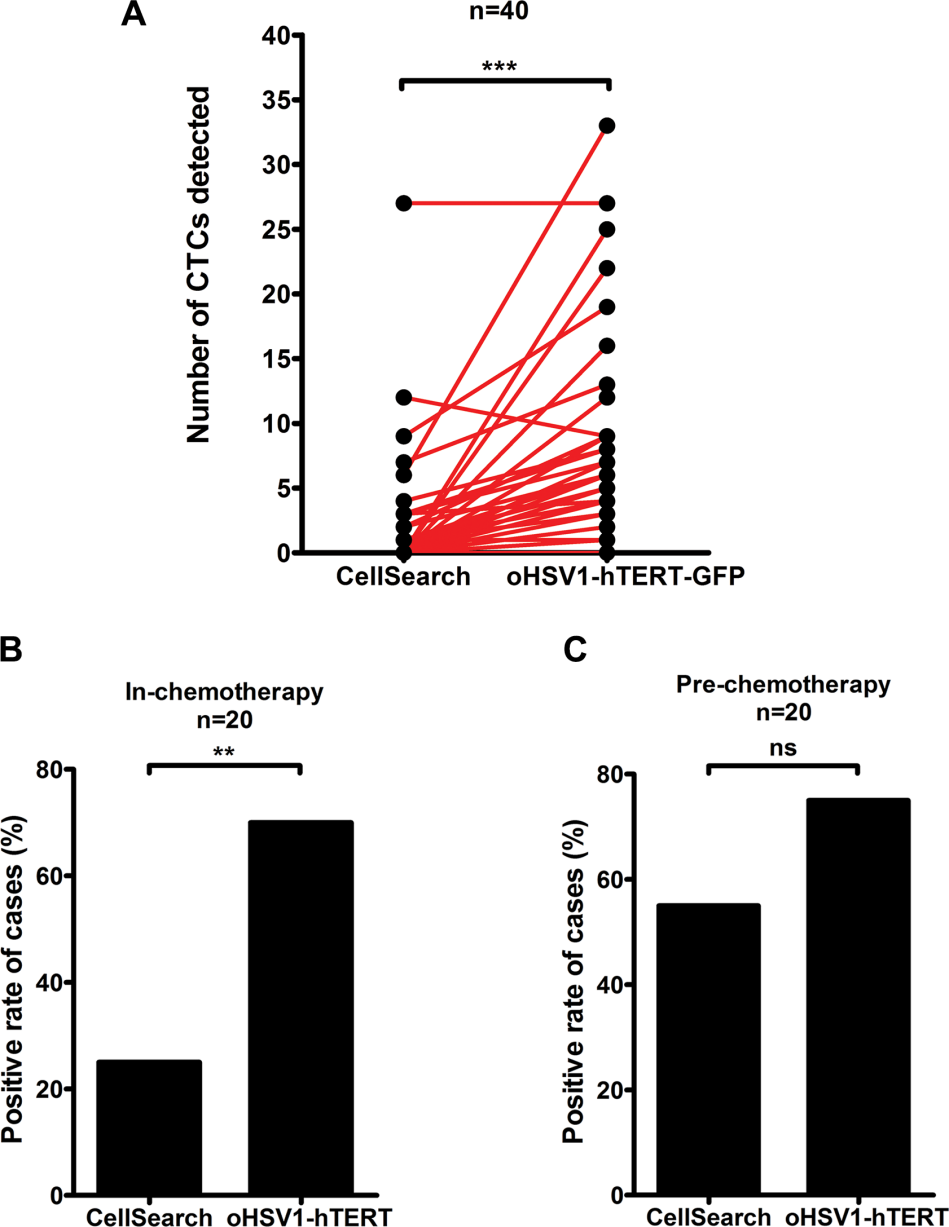
Comparison of CTC detection using oHSV1-hTERT-GFP and CellSearch methods (**A**) Detection of CTCs in peripheral blood of non–small cell lung cancer patients by CellSearch and oHSV1-hTERT-GFP. *n* = 40. (**B**–**C**) The CTC positive of NSCLC patients detected by CellSearch or oHSV1-hTERTp-GFP during chemotherapy treatment (B) or before chemotherapy treatment (C). ns, no significant difference, **p < 0.0005, ***p < 0.0001.

To compare our CTC detection method with that of CellSearch (Janssen Diagnostics, Raritan, NJ), blood samples from 40 lung adenocarcinoma patients were simultaneously analyzed using the two methods. The numbers of CTCs detected by oHSV1-hTERT-GFP ranged from 0 to 33 (Figure 7A, Supplementary Tables 14 and 15), while the numbers of CTCs detected by CellSearch ranged from 0 to 27. Higher CTC numbers were detected by oHSV1-hTERT-GFP vs. CellSearch, and CTC-positive rates were significant higher in the former (*p* < 0.0001). Similar results (positive or negative, respectively) were obtained by both methods in 57.5% of samples (23 of 40), while 35% (14 of 40) were only CTC-positive by oHSV1-hTERT-GFP. Of the 40 lung cancer patients, 20 had blood samples taken during chemotherapy, while the other 20 were obtained prior to chemotherapy. The CTC-positive rates of samples obtained during chemotherapy were 25% (5 to 20) for CellSearch and 70% (14 to 20) for oHSV1-hTERT-GFP, while CTC-positive rates for the samples obtained prior to chemotherapy were 55% (11 to 20) for CellSearch and 75% (15 to 20) for oHSV1-hTERT-GFP (Figure 7B and 7C). This indicates that cytotoxic systemic therapy had less impact on oHSV1-hTERT-GFP detection than on CellSearch in comparably staged patients. Interestingly, correlation of CTC-positive rates with TNM stages showed that oHSV1-hTERT-GFP was much more sensitive than CellSearch in lung cancers with regional lymph node metastasis or distant metastasis (Supplementary Table 16). The CTC-positive rates of N0M0 patients determined by pathology were 36.4% for CellSearch (4 of 11) and 54.5% for oHSV1-hTERT-GFP (6 of 11). The positive rates of N+M0 patients were 41.4% for CellSearch (12 of 29) and 75.9% for oHSV1-hTERT-GFP (22 of 29). Strikingly, CTCs for patients with distant metastasis were 100% positive (9 of 9) by oHSV1-hTERT-GFP vs 33.3% positive (3 of 9) by CellSearch. These observations strongly indicate that oHSV1-hTERT-GFP is a more sensitive assessment tool than CellSearch in lung cancer patients across the spectrum of disease.

## DISCUSSION

We established a reliable cell surface marker-independent approach to enumerate CTCs from as little as 4 ml of peripheral blood samples via tumor-specific HSV-transduced GFP. Our results show that this assessment methodology can more accurately determine CTC numbers to quantify circulating tumor cells, including EpCAM-negative CTCs, in patients with solid tumors, as well as identify CTCs in patients with hematological malignancies. More importantly, viable CTCs detected by this approach may serve as a reliable biomarker for monitoring tumor progression and therapeutic responses, making it a very useful clinical tool.

CTC detection methods with high sensitivity and specificity are in great demand for clinical application. Receiver operating characteristics (ROC) curve analysis of samples from 178 healthy donors and 290 patients with various solid tumors detected by oHSV1-hTERT-GFP generated a threshold of > 3 CTCs per 4 ml blood that differentiated cancer patients vs. healthy individuals. The threshold of hematological malignancies was 17 CTCs per 10^5^ cells based on ROC analysis. Our data confirmed the sensitivity of this method as well as the relatively high specificity for detection of CTCs from various cancer types.

Although efforts have been made to improve the accuracy and sensitivity of cancer diagnoses, earlier detection of metastasis received less attention. Our approach has shown the potential for early detection of micro residual disease, especially in patients with N0M0 stage. Interestingly, our data showed 50% of N0M0 patients also had detectable CTCs. Since current staging criteria for cancers primarily relies on information from pathology and surgical reports, micro-metastases are often overlooked with current standard histopathological techniques. It has been shown that 30–40% of patients with stage I NSCLC suffer post-operative local recurrence and distant metastasis despite pathologically-confirmed N0M0 [36, 37, 38], suggesting that the pathological stage defined by routine histopathology using lymph node cancers may underestimate rates of node-positive disease. Patients with early stage disease such as T0N0 should be followed in subsequent studies to clarify the relationship between the disease-free interval or survival with the number of CTCs in these subset of patients. CTCs from all patients with pathologically-determined distant metastases were almost uniformly positive when tested by our method except for a single pancreatic cancer patient in which CTCs may have been telomerase-negative or enter the blood stream intermittently. CTCs for patients with regional lymph node metastasis also showed > 70% positivity rates in all cancer types tested, indicating CTCs are common and thus detectable at this stage of cancer progression. Our data showed that oHSV1-hTERT-GFP effectively monitored patients with micro-metastasis, which would clearly assist clinicians in making more accurate judgments.

Our patient data showed that the number of viable CTCs accurately reflects the clinical response. Although the patient number in this study is limited, 6 hematological malignancy patients and 3 NSCLC patients exhibited a relevant decrease of CTC counts in response to chemotherapy, whereas a recrudescent hematological malignancy patient had a dramatically increased CTC count, validating the correlation of CTC numbers with disease progression. A sharp increase in CTC numbers thus indicates therapeutic failure or exacerbations during chemotherapy, suggesting that this approach may be even more valuable with occult disease and treatment response.

To date, a variety of methods have been established to isolate and enumerate CTCs, [13, 23, 39, 40, 41], but some shortcomings remain to be overcome. Our approach can be easily utilized in the absence of any tumor cell surface markers. CTCs can be identified and serially monitored whether patients have or have not received treatment, implying that oHSV1-hTERT-GFP is a reliable approach for CTC detection. In addition, combined with flow cytometry, our strategy allows a high-throughput detection methodology of a broad range of CTCs and isolation of single-cell CTCs for genomic analysis that will extend our current understanding of CTCs. Furthermore, CTCs under EMT or other biological changes due to novel mutations in the cancer cell genome may still be detected by our approach, which facilitates dynamic monitoring of CTCs for treatment response and tumor progression.

In conclusion, we have developed a robust HSV-based approach that is easily applicable in the clinic for accurate identification and sorting of viable CTCs from a broad spectrum of epithelial and non-epithelial malignancies. The approach has the potential to monitor tumor progression, predict prognosis, and evaluate therapeutic efficacy of individualized treatments. This is the first report showing that tumor-selective replicating HSV with GFP expression can be used for fast and robust detection of viable CTCs. Further, this method allows isolation of single CTCs for subsequent genetic and molecular analyses.

## MATERIALS AND METHODS

### Patient recruitment and sample collection

Eligible patients were diagnosed with hepatocellular carcinoma (HCC), gastric carcinoma, colon cancer, non–small cell lung cancer (NSCLC), glioma or lymphoma. The study was approved by the Cancer Institute & Hospital ethics committee. Clinical information of the included patients is provided in Supplementary Tables 5–12. All patients had metastatic solid cancers or hematological malignancies and were undergoing active treatment. The healthy donor age range was 20–50 years old. All patients provided written informed consent for collection of tissue and blood and analyses of clinical and genetic data for research purposes. Four ml blood was prospectively collected from eligible patients in heparinized tubes and transported on ice to the lab within 1 hour.

### Identification of samples with solid tumors

Peripheral blood samples (4 ml) were prepared in heparinized tubes and incubated with lysis buffer (NH_4_Cl, 0.15 M; EDTA, 0.1 mM; KHCO_3_, 10 mM; pH = 7.2) at RT for 5 minutes. After centrifugation, supernatant was discarded and cell pellets were washed 2× with PBS. Following centrifugation, cells were resuspended and transduced with oHSV1-hTERT-GFP at an MOI = 1 at 37°C in a humidified atmosphere of 5% CO_2_ for another 24 hours. Thereafter, the cells were gathered, 200 μl PE-Cy5 mouse anti-human CD45 (HI30, BD, USA) was added and incubated in the dark at room temperature for 30 minutes. After one wash with PBS, the cells were resuspended in 1 ml PBS. Detection of CD45−/GFP+ cells for solid tumor blood samples were executed by flow cytometry (Merck Millipore, Germany or BD, USA). The CD45−/GFP+ cells were recorded as positive results.

### Identification of CTCs with hematological malignancies

Peripheral blood samples (4 ml) were prepared in heparinized tubes and incubated with lysis buffer (NH_4_Cl, 0.15 M; EDTA, 0.1 mM; KHCO_3_, 10 mM; pH = 7.2) at RT for 5 minutes. After centrifugation, supernatant was discarded and cell pellets were washed x2 with PBS. Following centrifugation, cells were resuspended and transduced with oHSV1-hTERTp-GFP at an MOI = 1 at 37°C in a humidified atmosphere of 5% CO_2_ for another 24 hours. Thereafter, cells were gathered and detection of GFP+ cells for hematological malignancies was performed by flow cytometry (Merck Millipore, Germany or BD, USA). GFP+ cells of more than 17 were recorded as positive.

### Immunofluorescence analysis

Peripheral blood samples were pretreated as mentioned above. The cells were gathered after incubation with oHSV1-hTERT-GFP for 24 hours. Alexa Fluor^®^ 647 anti-Cytokeratin 18 antibody (DA-7, Biolegend, USA) was added and incubated in the dark at room temperature for 30 minutes. After one wash with PBS, the cells were resuspended in 1 ml PBS and CTCs were recorded by confocal microscopy (LEICA DMI RE2, Germany).

### Virus

The oHSV1-hTERT-GFP virus with the endogenous ICP4 promoter replaced with the hTERT promoter has been described in our previous work (42). The purified viruses were dissolved in SFM, titrated, divided into aliquots, and stored at −80°C until use.

### Cell culture

The tumor cell lines PC-3, HepG2 and MCF-7 were purchased from ATCC and maintained in our laboratory. The tumor cell lines HuH7, BEL-7402, SMMC-7721, PLC/PRF5, A549, HOS, and Wi-38 were purchased from the Cell Resource Center (IBMS, CAMS/PUMC). The BGC823 cell line was a gift from BGI-Shenzhen (Shenzhen, China). The human liver hepatocellular carcinoma cell lines HuH7, HepG2, BEL-7402, SMMC-7721 and PLC/PRF5, the human lung adenocarcinoma cancer cell line A549, the human gastric cancer cell line BGC823, the human osteosarcoma cell line HOS, the human prostate cancer cell line PC-3, and the human mammary gland adenocarcinoma cell line MCF-7 were cultured in DMEM/F12 medium (Invitrogen, USA) supplemented with 10% fetal bovine serum (FBS). The human glioma cell line U251 was cultured in MEM medium (Invitrogen, USA) supplemented with 10% FBS. The normal human fetal lung fibroblast cell line Wi-38 was cultured in MEM medium supplemented with 1% NEAA and 10% FBS. All cell lines were incubated at 37°C in a humidified atmosphere of 5% CO_2_ (Sanyo, Japan).

### Quantitative real-time RT-PCR

Total RNA was extracted from cultured cells using the TRIZOL reagent (Invitrogen, USA) according to manufacturer's instructions. The cDNA library was then reverse transcribed using the ReverTra Ace qPCR RT Master Mix kit (Toyobo, Japan) according to manufacturer's instructions. The following specific primers were used: hTERT, forward primer, 5′-CCGATT GTGAACATGGACTACG-3′ and reverse primer, 5′-CACG CTGAACAGTGCCTTC-3′; and GAPDH, forward primer, 5′-TGTGGGCATCAATGGATTTGG-3′ and reverse primer, 5′-ACACCATGTATTCCGGGTCAAT-3′. Quantitative RT-PCR (qRT-PCR) was performed using the SYBR Green I Mix kit (Toyobo, Japan) with a 7300 Real-Time PCR System (ABI, USA), as described. Each sample was run in triplicate, and the expression of each gene was presented as the ratio of the expression of the TERT mRNA and GAPDH mRNAs.

### GFP fluorescence intensity analysis

The cells were seeded in 6-well plates. After treatment with virus at an MOI of 0.1 for 48 h, the cells were harvested and analyzed by flow cytometry for GFP fluorescence intensity.

### Single-cell lysis and whole-genome amplification (WGA) of single cells

After isolation of single cells by flow cytometry, each cell was immediately transferred into a precooled PCR tube containing cell lysis solution on ice. A physiological saline blank was included as a negative control. WGA was performed using the REPLI-g Single Cell Kit according to the manufacturer's manual (Qiagen GmbH). All amplified DNA products were then stored at −20°C.

### Concentration measurements and whole-exome sequencing

The Qubit^™^ Quantitation Platform (Life Technologies) was used to measure concentrations of the WGA products. Thereafter, successfully amplified products with DNA yields reaching more than 1.5 μg were selected for further whole-exome sequencing using the SeqCap EZ Human Exome Library v3.0 (Roche NimbleGen) and HiSeq2000 platform (Illumina).

### Single-cell sequencing data analysis

Following whole-exome sequencing, reads were aligned to the NCBI human reference genome (hg19) using BWA (v0.5.9), duplicates were removed by Picard (v1.54), and calling of SNPs was performed using the Genome Analysis Tool Kit pipeline. SNPs detected in the amplification products of the single-cell samples were filtered by the quality cutoff value of 50 for comparison with SNPs of the SMMC-7721 cell line and the peripheral blood of the healthy donor to identify the source of the cells. To avoid random deviations of calling SNPs, we selected regions where the SMMC-7721 cell line and peripheral blood of the healthy donor both covered with depth greater than 30×.

### Statistics

All quantitative data are reported as the mean ± SED. Statistical analysis was performed for multiple comparisons using analysis of variance, Student's *t*-tests, and Chi-square test. The accuracy of the detection method and the association between bio-markers and number of CTCs was analyzed using linear regression as appropriate. The SPSS 15 software package (SPSS, Inc.) was used for ROC analysis. A *P* value < 0.05 was considered statistically significant.

### Study approval

All protocols involving patients were approved by the Cancer Institute & Hospital ethics committee.

## SUPPLEMENTARY MATERIALS FIGURES AND TABLES






