# Supplementary material for: Tumor-selective replication herpes simplex virus-based technology significantly improves clinical detection and prognostication of viable circulating tumor cells
Source: Oncotarget. 2016 May 18;7(26):39768–83. doi: 10.18632/oncotarget.9465 (PMC5129969; doi:10.18632/oncotarget.9465)
Supplement: Supplementary file 1 [file oncotarget-07-39768-s001.pdf]

Tumor-selective replication herpes simplex virus-based technology significantly improves clinical detection and prognostication of viable circulating tumor cells

Supplementary Materials

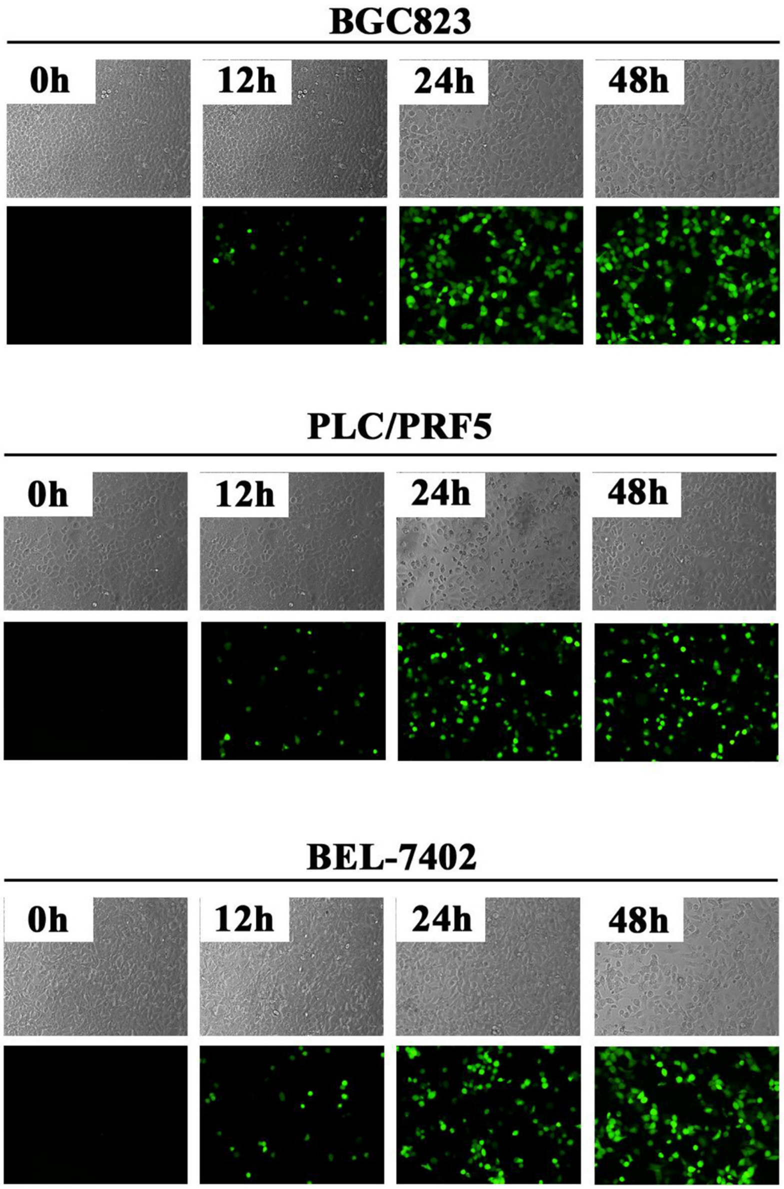

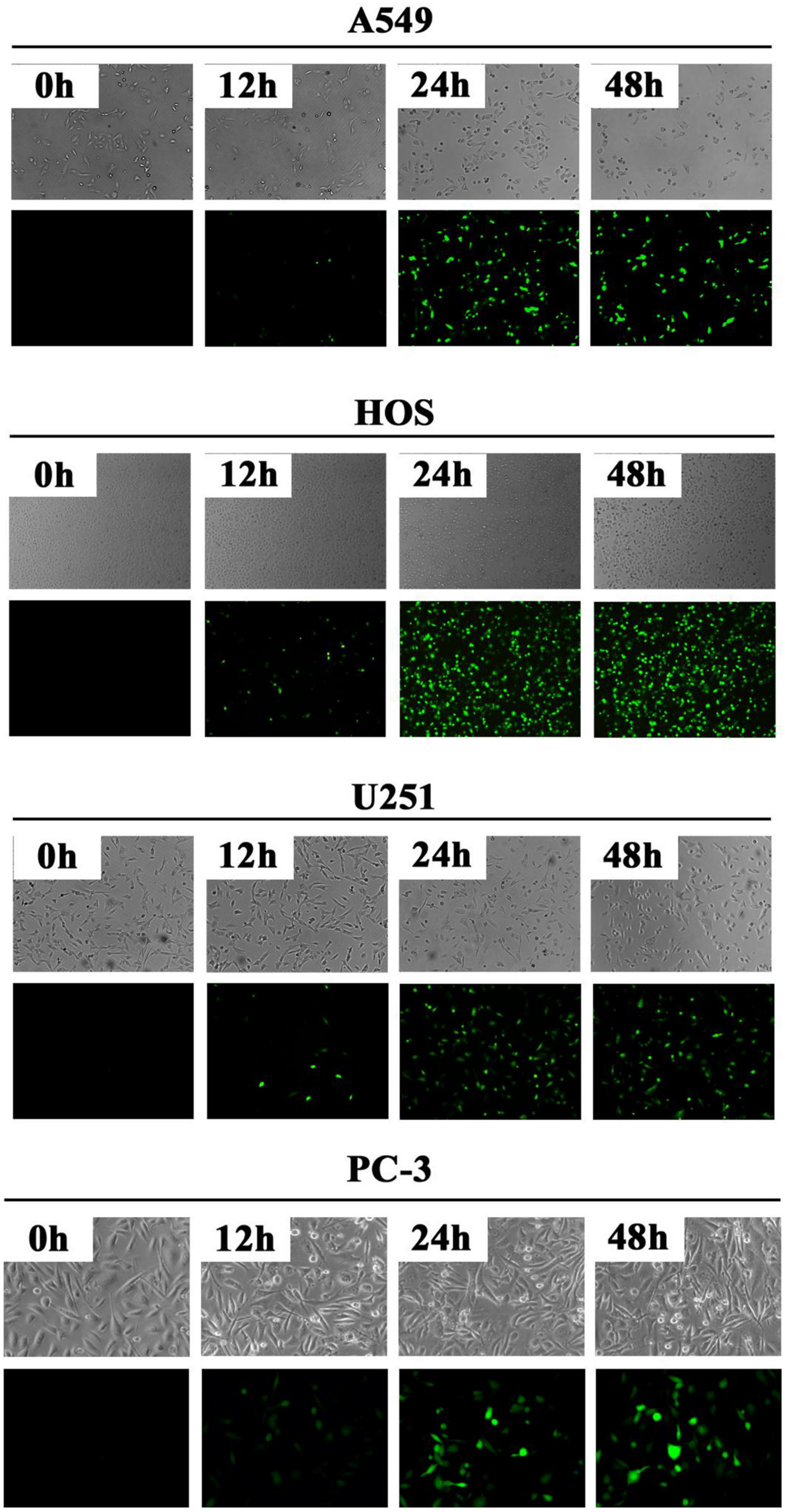

Supplementary Figure S1: Images of BGC823, PLC/PRF5, BEL-7402, A549, HOS, U251 and PC3 cells were recorded for 48 hours after oHSV1-hTERTp-GFP transduction at an MOI of 1. Selected images taken at the indicated time points show cell morphology by phase-contrast microscopy (top panels) and GFP expression by fluorescence microscopy (bottom panels). Original magnification, ×100 for BGC823, PLC/PRF5, BEL-7402, A549, HOS, and U251 cells. Original magnification, ×200 for PC3 cells.

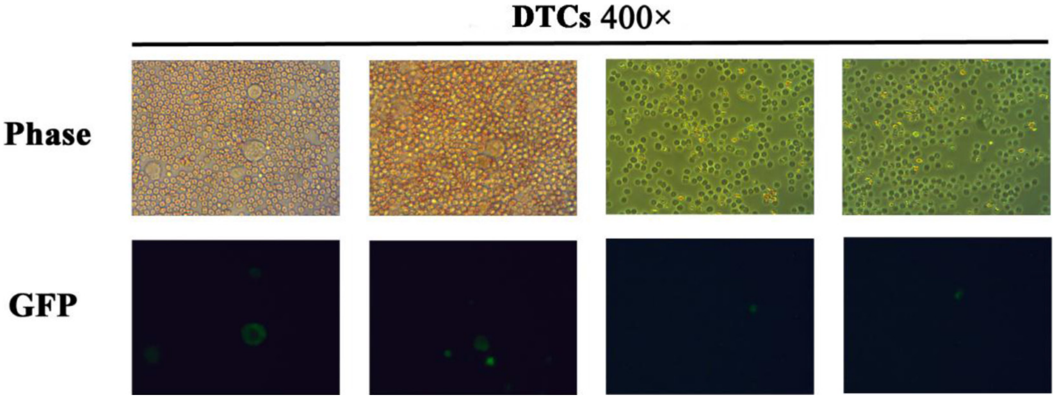

Supplementary Figure S2: Typical DTCs in the cerebrospinal fluid of glioma patients were visualized using GFP expression. Original magnification, ×400.

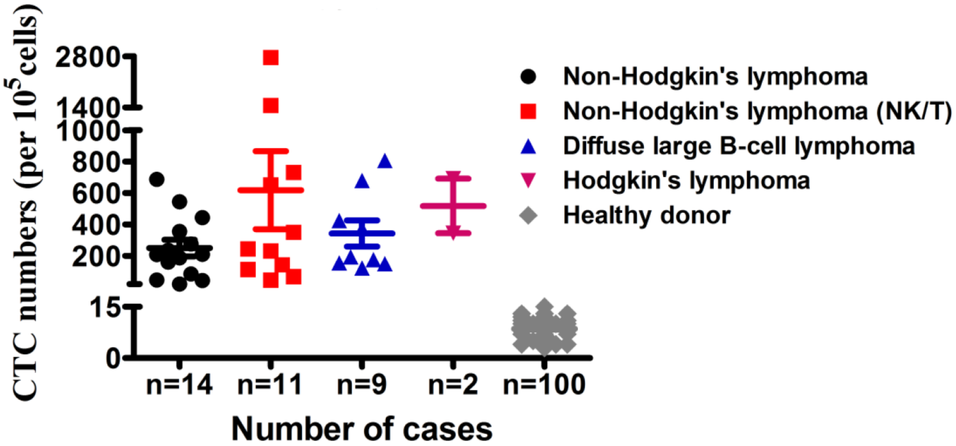

Supplementary Figure S3: Enumeration of CTCs in peripheral blood of hematological malignancy patients, CTC counts in 4-ml blood samples from 14 patients with Non-Hodgkin's lymphoma, 11 with Non-Hodgkin's lymphoma (NK/T), 9 with diffuse large B-cell lymphoma, and 2 with Hodgkin's lymphoma.

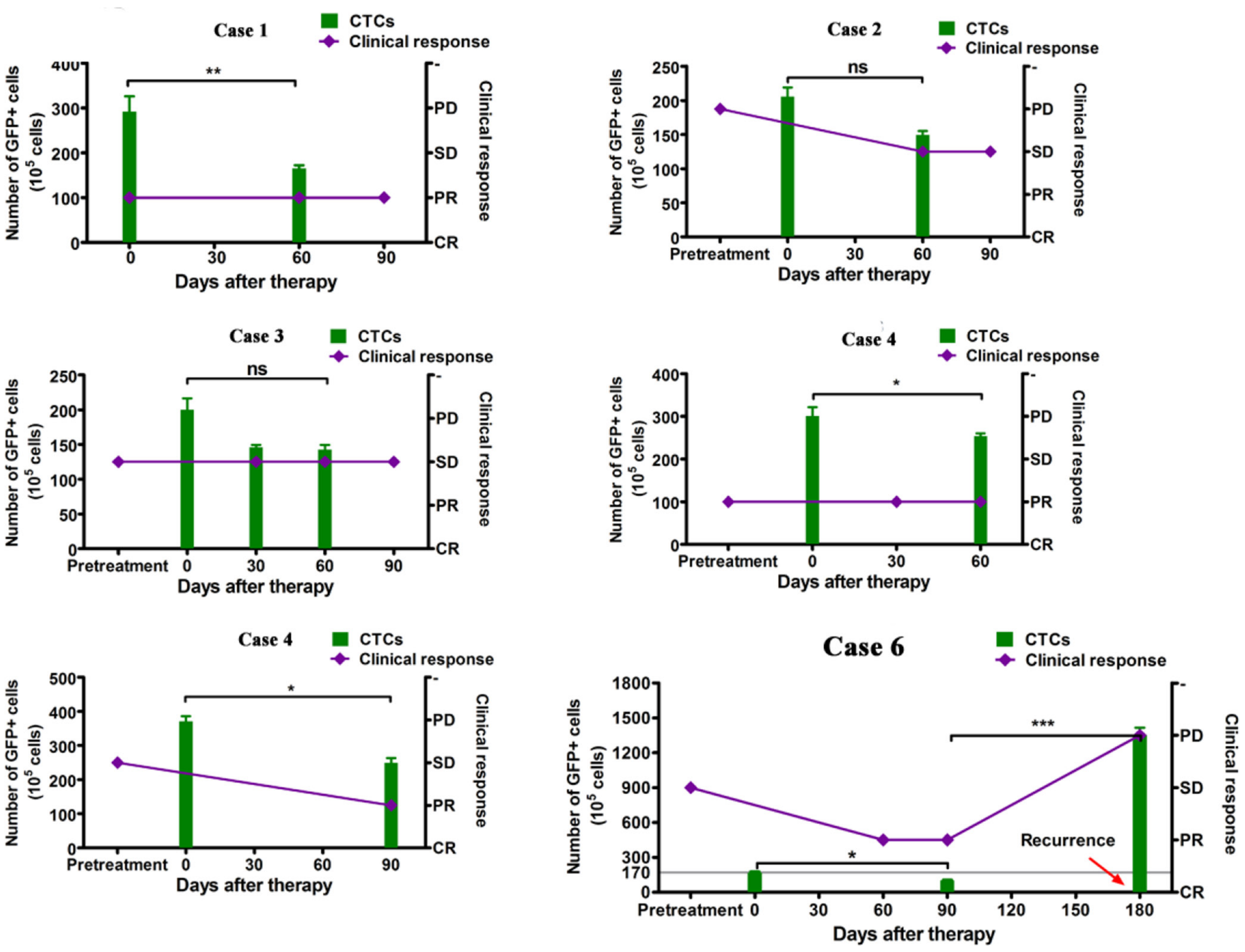

Supplementary Figure S4: Enumeration of GFP+ CTCs in the peripheral blood of patients with lymphoma. Number of CTCs in peripheral blood samples from 5 patients (case 1 from 292 to 165, case 2 from 206 to 150, case 3 from 200 to 142, case 4 from 300 to 253, and case 5 from 371 to 249) with lymphoma who received 2 cycles of chemotherapy. Changes in the CTCs in peripheral blood samples from case 6 with Non-Hodgkin's lymphoma. CTC counts at the indicated time points (green bars) along with the evaluation of curative effect were plotted. A boost in the average CTC number from 102 to 1354 per 105 cells was observed 90 days after 2 cycles of chemotherapy. Average CTC counts at the indicated time points (green bars) were plotted along with the evaluation of clinical response (*$p < 0.05$; **$p < 0.01$; ns, no significant differences). PD, progressive disease; SD, stable disease; PR, partial response; CR, complete response.

Supplementary Table S1: Recovery rate of BGC823 cells detected by oHSV1-hTERTp-GFP

Number of tumor cells	0	10	20	40	80	160	320
Mean	0.35	7.55	15.27	31.44	64.14	130.29	279
Detection rate (%)	-	75.5%	76.33%	78.61%	80.18%	81.42%	87.19%
Number of samples	20	20	15	10	8	8	5

Supplementary Table S2: Recovery rate of HuH7 cells detected by oHSV1-hTERTp-GFP

Number of tumor cells	0	10	50	100
Mean	0.35	7.5	41.8	88.4
Detection rate (%)	-	75%	83.6%	88.4%
Number of samples	20	10	5	5

Supplementary Table S3: Recovery rate of SMMC-7721 cells detected by oHSV1-hTERTp-GFP

Number of tumor cells	0	10	50	100
Mean	0.35	7.2	42	88.2
Detection rate (%)	-	72%	84%	88.2%
Number of samples	20	10	5	5

Supplementary Table S4: Summary of data obtained by single-cell sequencing

Sample name	Data size (M)	Mean depth	M of bases at ≥ 1X depth	% of bases at ≥ 1X depth	Duplication rate (%)	GC content (%)
GFP-P1	911	7.0	48.00	75.00	3.60	44.61
GFP-P2	509	4.9	41.65	65.08	3.01	45.19
GFP-P3	567	5.4	49.00	76.56	2.98	45.27
GFP-P4	1310	12.9	33.60	52.50	6.48	45.69
GFP-P5	495	4.7	39.50	61.72	2.70	44.67
GFP-P6	673	6.4	42.80	66.88	3.30	46.52
GFP-N1	574	5.5	53.10	82.97	3.18	44.89
GFP-N2	672	6.5	55.70	87.03	3.20	46.2
GFP-N3	870	5.8	43.40	67.81	3.24	44.35
GFP-NP	2850	21.6	44.84	79.06	5.85%	44.6
PB	6300	60.7	62.10	97.03	4.28	45.5
iSMMC	4700	43.8	61.75	96.48	7.32	46.08

Supplementary Table S5: Characteristics and CTC numbers in non-small cell lung cancer patients. See Supplementary_Table_S5

Supplementary Table S6: Characteristics and CTC numbers in small cell lung cancer patients

No	Age	Gender	Stage	CTCs
1	M	44	ED	7
2	F	70	ED	5
3	M	72	ED	0
4	M	64	LD	1
5	M	56	LD	9
6	F	36	ED	33
7	M	37	LD	2
8	M	56	ED	21
9	F	61	ED	6
10	M	75	ED	9
11	M	62	ED	7
12	M	60	LD	2
13	F	40	ED	17
14	M	56	ED	3

Supplementary Table S7: Characteristics and CTC numbers in colorectal cancer patients. See Supplementary_Table_S7

Supplementary Table S8: Characteristics and CTC numbers in gastric cancer patients

No	Age	Gender	TNM	CTCs
1	71	M	T4aN1M0	8
2	54	F	T4bN2M0	12
3	49	M	T4N2M1	7
4	63	M	T3N0M0	2
5	56	F	T3N3M0	25
6	64	F	T2N3M0	8
7	72	M	T4bN3M0	18
8	46	M	T1N3M0	7
9	33	M	T3N1M0	11
10	68	M	T4bN1M0	15
11	51	M	T4aN2M1	43
12	61	F	T3N3M1	15
13	58	M	T4bN1M0	8
14	46	F	T4aN3M0	15
15	73	F	T4N3M1	28
16	69	M	T1N2M0	1
17	33	M	T3N3M0	2
18	42	F	T1N2M0	4
19	60	F	T4bN1M0	23
20	42	M	T1N3M0	12
21	53	F	T4aN1M0	13
22	58	M	T4N3M1	7
23	57	F	T4N3M1	4
24	49	F	T2N3M0	27
25	66	F	T3N3M0	17
26	64	M	T4N3M1	71
27	52	M	T3N2M0	3
28	61	F	T4bN0M0	5
29	57	M	T4N3M1	37

Supplementary Table S9: Characteristics and CTC numbers in glioma patients

No	Age	Gender	Grade	CTCs
1	43	F	A II	0
2	8	F	IV	4
3	47	M	A II	11
4	29	F	A II-III	3
5	23	M	AO III	5
6	29	M	OA II-III	8
7	50	F	AII	3
8	57	M	IV	3
9	44	M	OA II	3
10	41	F	A II-III	2
11	51	M	O II	2
12	62	F	AO III	2
13	57	F	IV	6
14	43	F	IV	13
15	65	M	I	2
16	25	M	I	4
17	63	F	IV	8
18	58	F	IV	6
19	54	F	IV	12
20	41	M	IV	18
21	30	M	AOA III	18
22	49	F	IV	5
23	42	M	IV	20
24	20	M	IV	7
25	65	M	IV	2
26	64	M	AO III	3
27	41	M	AO III	8
28	38	M	OA II-III	2
29	37	M	O II	3
30	48	F	IV	2
31	58	F	O II	0
32	56	M	II	14
33	72	F	AO III	2
34	45	M	OA II	5
35	40	F	IV	5
36	61	F	AOA III-IV	8
37	75	M	AOA III	8
38	44	F	O II	17
39	39	M	OA II-III	9

*A: Astrocytoma; O: Oligodendroglioma; OA: Oligoastrocytomas; AO: Anaplastic oligodendroglioma; AOA: Anaplastic oligoastrocytomas.

Supplementary Table S10: Characteristics and CTC numbers in hepatocellular carcinoma patients

No	Age	Gender	CTCs	TNM	AFP (ng/ml)
1	66	F	9	T1NxM0	1.44
2	66	M	23	T2N1M0	430
3	67	M	17	T1N1M0	3.74
4	63	M	4	T2N1M0	2.68
5	54	M	25	T3N1M1	2.53
6	63	F	18	T1N0M0	4.07
7	30	F	48	T4N1M1	1210
8	73	M	32	T3N0M0	704.2
9	69	M	16	T2N1M0	420
10	54	M	2	T1N1M0	1.34
11	45	F	18	T3N0M0	312.5
12	53	M	23	T4NxM0	4.32
13	48	F	17	T2N0M0	152.8
14	52	F	3	T2N0M0	5.26
15	61	F	15	T3N1M0	680
16	48	M	4	T2N0M0	132
17	47	M	8	T3N0M0	7.68
18	54	F	7	T2N0M0	5.56
19	62	F	24	T3N1M1	670.3
20	49	F	13	T3N0M0	157
21	51	F	33	T3N1M1	621.5
22	73	F	27	T2N0M0	11.2
23	66	M	13	T2N0M0	220
24	57	F	3	T2N0M0	1.07
25	48	M	8	T2N1M0	5.24
26	47	M	7	T3N0M0	1.23
27	48	F	51	T4N1M1	706.8
28	57	F	6	T1N1M1	3.62
29	29	M	6	T3N0M0	10.34
30	75	M	15	T2N0M0	2.68
31	65	M	54	T4N1M1	5.84
32	58	F	13	T1NxM0	2.78
33	46	F	6	T1N1M0	3.12
34	53	F	39	T2N1M1	9.69
35	48	F	8	T2NxM0	21.22
36	61	M	39	T1N1M0	10.13

Supplementary Table S11: Characteristics and CTC numbers in pancreatic cancer patients

No	Age	Gender	TNM	CTCs
1	M	52	T3N1M0	4
2	M	48	T4N0M0	4
3	M	62	T4N1M0	6
4	F	43	T4N1M1	41
5	M	71	T4N0M0	13
6	M	58	T4N0M0	1
7	M	44	T4N1M1	22
8	F	51	T4N1M1	25
9	M	64	T4N0M1	28
10	M	42	T4N0M1	3
11	M	47	T4N1M1	19
12	M	55	T4N1M1	11
13	M	62	T4N0M1	33
14	M	59	T4N1M1	194
15	F	62	T4N1M1	99
16	M	53	T4N1M1	130
17	M	49	T4N1M1	100

Supplementary Table S12: Characteristics and CTC numbers in hematological malignancy patientss

Type	No	Age	Gender	Stage	CTCs
Non-Hodgkin's lymphoma	1	64	M	IV A	208
	2	41	M	III	355
	3	64	M	IV B	544
	4	59	M	II A	19
	5	35	M	IV A	43
	6	14	F	IV A	84
	7	76	F	II A	45
	8	62	M	III A	235
	9	65	M	IV A	209
	10	16	M	IV	687
	11	41	M	IV B	275
	12	27	M	II B	159
	13	33	F	II EB	187
	14	31	M	III BX	443
Non-Hodgkin's lymphoma (NK/T)	1	30	M	IV A	2,767
	2	45	M	I EB	68
	3	25	F	III AX	350
	4	36	F	IV A	144
	5	36	F	II BX	1456
	6	58	M	III A	112
	7	47	F	IV A	731
	8	28	F	IV A	651
	9	52	F	I EB	46
	10	32	M	IV B	244
	11	47	M	IV EB	231
Diffuse large B-cell lymphoma	1	42	M	IV A	423
	2	49	F	II EA	678
	3	62	F	III A	807
	4	55	M	III A	193
	5	49	M	IV A	373
	6	61	F	II A	121
	7	56	M	II A	155
	8	75	M	IV A	150
	9	33	M	IV AX	176
Hodgkin lymphoma	1	36	F	III A	691
	2	32	M	II BX	343

Supplementary Table S13: Detection of CTC numbers in non-small cell lung cancer (adenocarcinoma) patients pre- and post-chemotherapy

Case No	Pre-Chemotherapy	Post-Chemotherapy
5	6	2
10	1	7
11	16	10
12	25	6
13	7	4
14	22	5
15	8	1
20	12	20
25	8	4
27	3	20
29	33	9
31	14	4
35	49	5
39	6	15
40	14	5
47	25	2
48	19	3
53	14	5
56	53	24
59	15	9
66	14	6

Supplementary Table S14: Detection of CTC numbers in non-small cell lung cancer (adenocarcinoma) patients by CellSearch and oHSV1-hTERT-GFP before chemotherapy

No	Cell Search	oHSV	TMN
1	7	13	T3N1M0
2	2	6	T3N3M1
3	0	4	T3N0M0
4	1	3	T2bN1M0
5	4	8	T3N1M0
6	27	27	T2N2M1
7	0	3	T2N1M0
8	3	7	TxN3M0
9	6	33	T2aN1M1
10	0	5	T4N2M0
11	0	3	T2bN1M0
12	3	4	T3N0M0
13	12	9	T4N1M0
14	1	5	T4N1M0
15	0	4	T2bN2M1
16	0	1	T2bN0M0
17	9	19	T2bN0M0
18	0	3	T4N0M0
19	0	1	T2bN0M0
20	0	6	T3N2M0

Supplementary Table S15: Detection of CTC numbers in non-small cell lung cancer (adenocarcinoma) patients by CellSearch and oHSV1-hTERT-GFP during chemotherapy

No	Cell Search	oHSV	TMN
1	1	1	T2bN0M0
2	1	5	T3N0M0
3	0	4	T2aN1M1
4	0	2	T3N0M0
5	0	6	T4N2M0
6	0	4	T2bN2M1
7	0	2	TxN2M0
8	0	25	T2bN0M0
9	0	2	T1N1M0
10	0	1	T2aN1M0
11	0	16	T3N1M1
12	0	25	T2bN2M1
13	0	7	T1N0M0
14	0	22	T2bN3M1
15	3	8	T2bN1M0
16	1	6	T3N2M0
17	0	0	T2aN1M0
18	0	9	T2bN1M0
19	2	9	T2aN1M0
20	0	12	T3N2M0

Supplementary Table S16: Comparison of sensitivity of CTCs detection in non-small cell lung cancer (adenocarcinoma) patients by CellSearch and oHSV1-hTERT-GFP

Type	TNM	Number of negative patients	Total number of patients	Sensitivity*
CellSearch	T1N0M0	1	1	0
	N0 M0	7	11	0.364
	N+ M0	11	20	0.45
	N+M1	6	9	0.333
oHSV1-hTERT-GFP	T1N0M0	0	1	1
	N0M0	5	11	0.545
	N+M0	7	20	0.65
	N+M1	0	9	1

Note: * Sensitivity =1-(number of negative patients/total number of patients).
