# Supplementary material for: Tumor-selective replication herpes simplex virus-based technology significantly improves clinical detection and prognostication of viable circulating tumor cells
Source: Oncotarget. 2016 May 18;7(26):39768–83. doi: 10.18632/oncotarget.9465 (PMC5129969; doi:10.18632/oncotarget.9465)
Supplement: Supplementary file 2 [file oncotarget-07-39768-s002.docx]

Supplementary Table 5: Characteristics and CTC numbers in non-small cell lung cancer patients

| Type | | No | | Age | | Gender | | TNM | | CTCs | |
| --- | --- | --- | --- | --- | --- | --- | --- | --- | --- | --- | --- |
| Adenocarcinoma | | 1 | | 61 | | M | | T2bN0M0 | | 1 | |
|  |  | 2 | | 76 | | M | | T3N0M0 | | 5 | |
|  |  | 3 | | 63 | | M | | T2aN1M1 | | 4 | |
|  |  | 4 | | 62 | | M | | T3N0M0 | | 2 | |
|  |  | 5 | | 65 | | M | | T4N2M0 | | 6 | |
|  |  | 6 | | 65 | | M | | T2bN2M1 | | 4 | |
|  |  | 7 | | 50 | | M | | TxN2M0 | | 2 | |
|  |  | 8 | | 63 | | M | | T2bN0M0 | | 25 | |
|  |  | 9 | | 44 | | M | | T1N1M0 | | 2 | |
|  |  | 10 | | 62 | | M | | T2aN1M0 | | 1 | |
|  |  | 11 | | 37 | | F | | T3N1M1 | | 16 | |
|  |  | 12 | | 57 | | F | | T2bN2M1 | | 25 | |
|  |  | 13 | | 60 | | M | | T1N0M0 | | 7 | |
|  |  | 14 | | 46 | | M | | T2bN3M1 | | 22 | |
|  |  | 15 | | 53 | | M | | T2bN1M0 | | 8 | |
|  |  | 16 | | 64 | | M | | T3N2M0 | | 6 | |
|  |  | 17 | | 51 | | F | | T2aN1M0 | | 0 | |
|  |  | 18 | | 50 | | M | | T2bN1M0 | | 9 | |
|  |  | 19 | | 51 | | M | | T2aN1M0 | | 9 | |
| Adenocarcinoma | 20 | | 60 | | M | | T3N2M0 | | 12 | |  |
|  | 21 | | 42 | | M | | T3N1M0 | | 13 | |  |
|  | 22 | | 58 | | M | | T3N3M1 | | 6 | |  |
|  | 23 | | 71 | | M | | T3N0M0 | | 4 | |  |
|  | 24 | | 42 | | M | | T2bN1M0 | | 3 | |  |
|  | 25 | | 47 | | F | | T3N1M0 | | 8 | |  |
|  | 26 | | 45 | | F | | T3N2M1 | | 27 | |  |
|  | 27 | | 63 | | F | | T3N1M0 | | 3 | |  |
|  | 28 | | 52 | | M | | TxN3M0 | | 7 | |  |
|  | 29 | | 55 | | M | | T2aN1M1 | | 33 | |  |
|  | 30 | | 46 | | M | | T2bN1M0 | | 3 | |  |
|  | 31 | | 68 | | M | | T3N1M0 | | 14 | |  |
|  | 32 | | 67 | | F | | T2aN0M0 | | 22 | |  |
|  | 33 | | 38 | | F | | T1N2M0 | | 5 | |  |
|  | 34 | | 57 | | M | | T3N0M0 | | 8 | |  |
|  | 35 | | 49 | | F | | T3N3M1 | | 49 | |  |
|  | 36 | | 62 | | F | | T4N2M0 | | 5 | |  |
|  | 37 | | 61 | | M | | T4N3M0 | | 51 | |  |
|  | 38 | | 38 | | M | | T3N1M0 | | 4 | |  |
|  | 39 | | 48 | | M | | T3N2M0 | | 6 | |  |
|  | 40 | | 46 | | M | | T4N1M0 | | 14 | |  |
|  | 41 | | 52 | | F | | T3N3M0 | | 5 | |  |
|  | 42 | | 56 | | M | | T2bN3M0 | | 13 | |  |
|  | 43 | | 63 | | F | | T4N1M0 | | 9 | |  |
|  | 44 | | 50 | | M | | T3N0M0 | | 9 | |  |
|  | 45 | | 56 | | M | | T4N2M1 | | 7 | |  |
|  | 46 | | 40 | | F | | T4N2M0 | | 7 | |  |
|  | 47 | | 42 | | M | | T3N3M1 | | 25 | |  |
|  | 48 | | 51 | | F | | T2bN0M0 | | 19 | |  |
|  | 49 | | 78 | | M | | T3N0M0 | | 9 | |  |
|  | 50 | | 37 | | M | | T1N3M0 | | 7 | |  |
|  | 51 | | 48 | | F | | T2bN3M0 | | 9 | |  |
|  | 52 | | 58 | | M | | T3N1M1 | | 16 | |  |
|  | 53 | | 56 | | F | | T3N2M1 | | 14 | |  |
|  | 54 | | 72 | | M | | T2aN0M0 | | 3 | |  |
|  | 55 | | 45 | | M | | T4N2M0 | | 40 | |  |
|  | 56 | | 63 | | M | | T4N2M1 | | 53 | |  |
|  | 57 | | 61 | | M | | T3N0M0 | | 7 | |  |
|  | 58 | | 75 | | M | | T3N2M0 | | 14 | |  |
|  | 59 | | 44 | | M | | T1N0M0 | | 15 | |  |
|  | 60 | | 69 | | F | | T4N1M0 | | 5 | |  |
|  | 61 | | 62 | | M | | T4N0M0 | | 2 | |  |
|  | 62 | | 61 | | M | | T2aN3M0 | | 1 | |  |
|  | 63 | | 40 | | M | | T1N0M0 | | 0 | |  |
|  | 64 | | 54 | | F | | T2bN3M0 | | 5 | |  |
|  | 65 | | 63 | | F | | T2aN0M0 | | 1 | |  |
|  | 66 | | 56 | | M | | T3N1M1 | | 14 | |  |
|  | 67 | | 47 | | M | | T4N0M0 | | 3 | |  |
|  | 68 | | 39 | | M | | T2bN0M0 | | 1 | |  |
| Squamous carcinoma | 1 | | 45 | | M | | T3N2M0 | | 14 | |  |
|  | 2 | | 60 | | M | | T3N3M1 | | 10 | |  |
|  | 3 | | 54 | | M | | T3N0M0 | | 2 | |  |
|  | 4 | | 67 | | F | | T4N2M0 | | 27 | |  |
|  | 5 | | 52 | | F | | T3N2M0 | | 0 | |  |
|  | 6 | | 71 | | M | | T2bN1M0 | | 6 | |  |
|  | 7 | | 66 | | M | | T4N2M1 | | 20 | |  |
|  | 8 | | 61 | | M | | T3N2M0 | | 4 | |  |
|  | 9 | | 51 | | F | | T2aN1M0 | | 5 | |  |
|  | 10 | | 46 | | M | | T4N1M0 | | 4 | |  |
|  | 11 | | 64 | | M | | T4N2M0 | | 5 | |  |
|  | 12 | | 66 | | F | | T4N1M1 | | 12 | |  |
|  | 13 | | 40 | | M | | T2bN1M0 | | 32 | |  |
|  | 14 | | 49 | | M | | T1N0M0 | | 1 | |  |
|  | 15 | | 56 | | F | | T2bN1M0 | | 7 | |  |
|  | 16 | | 35 | | F | | T3N0M0 | | 4 | |  |
|  | 17 | | 47 | | F | | T2aN1M1 | | 5 | |  |
|  | 18 | | 64 | | M | | T3N2M1 | | 15 | |  |
|  | 19 | | 62 | | M | | T2bN0M0 | | 7 | |  |
