# Supplementary material for: Tumor-selective replication herpes simplex virus-based technology significantly improves clinical detection and prognostication of viable circulating tumor cells
Source: Oncotarget. 2016 May 18;7(26):39768–83. doi: 10.18632/oncotarget.9465 (PMC5129969; doi:10.18632/oncotarget.9465)
Supplement: Supplementary file 3 [file oncotarget-07-39768-s003.docx]

Supplementary Table 7: Characteristics and CTC numbers in colorectal cancer patients

| No | Age | Gender | TNM | CTCs |
| --- | --- | --- | --- | --- |
| 1 | 61 | M | T2N1M0 | 13 |
| 2 | 66 | M | T1N2aM0 | 2 |
| 3 | 63 | F | T3N1M1a | 16 |
| 4 | 42 | F | T4bN0M0 | 9 |
| 5 | 56 | M | T3N2aM0 | 16 |
| 6 | 61 | F | T1N2bM0 | 3 |
| 7 | 58 | M | T4bN2M0 | 7 |
| 8 | 56 | M | T1N1cM0 | 3 |
| 9 | 62 | M | T4aN2aM1b | 33 |
| 10 | 61 | M | T1N2aM0 | 3 |
| 11 | 46 | F | T3N1M0 | 0 |
| 12 | 61 | M | T4aN2bM0 | 14 |
| 13 | 75 | M | T3N1M1a | 33 |
| 14 | 44 | F | T1N2aM0 | 15 |
| 15 | 39 | M | T4aN2aM1b | 13 |
| 16 | 55 | F | T3N2bM0 | 3 |
| 17 | 62 | F | T4aN2aM1b | 44 |
| 18 | 53 | M | T2N2bM0 | 1 |
| 19 | 65 | M | T1N2bM0 | 4 |
| 20 | 75 | F | T4bN2M0 | 50 |
| 21 | 43 | M | T4aN1M0 | 5 |
| 22 | 58 | M | T1N2aM0 | 23 |
| 23 | 54 | M | T4aN0M0 | 4 |
| 24 | 63 | F | T1N2bM0 | 10 |
| 25 | 56 | F | T3N2M1a | 6 |
| 26 | 71 | M | T4aN2bM0 | 41 |
| 27 | 54 | F | T4aN1M1a | 13 |
| 28 | 49 | M | T4bN0M0 | 1 |
| 29 | 63 | F | T3N1M1a | 29 |
| 30 | 56 | M | T4bN0M0 | 2 |
| 31 | 52 | M | T4aN0M0 | 4 |
| 32 | 46 | F | T3N1M1a | 68 |
| 33 | 37 | M | T1N2aM0 | 2 |
| 34 | 64 | F | T3N2aM1b | 63 |
| 35 | 43 | M | T4aN2aM0 | 0 |
| 36 | 66 | M | T1N2aM0 | 3 |
| 37 | 57 | M | T2N2bM0 | 28 |
| 38 | 52 | M | T2N0M0 | 0 |
| 39 | 48 | M | T2N1M0 | 19 |
| 40 | 68 | F | T1N2bM0 | 9 |
| 41 | 64 | F | T3N2bM0 | 4 |
| 42 | 58 | M | T4bN2bM0 | 14 |
| 43 | 51 | F | T4aN2aM0 | 36 |
| 44 | 63 | M | T2N2bM1a | 7 |
| 45 | 61 | F | T1N2aM0 | 13 |
| 46 | 38 | M | T3N2aM1a | 16 |
| 47 | 48 | M | T3N1M1b | 50 |
| 48 | 55 | F | T3N2bM0 | 14 |
| 49 | 62 | F | T4aN2aM1a | 14 |
| 50 | 56 | M | T3N1M0 | 2 |
| 51 | 64 | M | T3N1M1a | 7 |
| 52 | 50 | M | T4bN0M0 | 6 |
| 53 | 68 | M | T4aN2bM1 | 35 |
| 54 | 71 | F | T3N1M1a | 17 |
| 55 | 42 | M | T3N1M1a | 6 |
| 56 | 51 | M | T3N2aM0 | 12 |
| 57 | 78 | F | T2N2bM0 | 25 |
| 58 | 51 | M | T1N1cM0 | 3 |
| 59 | 56 | F | T3N1M1a | 8 |
| 60 | 40 | M | T2N2bM1a | 12 |
| 61 | 46 | M | T2N2aM0 | 0 |
| 62 | 52 | F | T4bN1M0 | 26 |
| 63 | 56 | F | T1N2bM0 | 6 |
| 64 | 63 | M | T4aN2aM1a | 22 |
| 65 | 56 | M | T4aN2M1a | 27 |
| 66 | 68 | F | T4aN0M0 | 0 |
| 67 | 47 | M | T2N2bM0 | 6 |
| 68 | 62 | F | T2N2bM0 | 18 |
